# Mohawk promotes the maintenance and regeneration of the outer annulus fibrosus of intervertebral discs

**DOI:** 10.1038/ncomms12503

**Published:** 2016-08-16

**Authors:** Ryo Nakamichi, Yoshiaki Ito, Masafumi Inui, Naoko Onizuka, Tomohiro Kayama, Kensuke Kataoka, Hidetsugu Suzuki, Masaki Mori, Masayo Inagawa, Shizuko Ichinose, Martin K. Lotz, Daisuke Sakai, Koichi Masuda, Toshifumi Ozaki, Hiroshi Asahara

**Affiliations:** 1 Department of Systems Biomedicine, Tokyo Medical and Dental University, 1-5-45 Yushima, Bunkyo-ku, Tokyo 113-8510, Japan; 2 Department of Orthopaedic Surgery, Okayama University Graduate School of Medicine, Dentistry, and Pharmaceutical Sciences, 2-5-1 Shikata-cho, Kita-ku, Okayama 700-8558, Japan; 3 Department of Systems Biomedicine, National Research Institute for Child Health and Development, 2-10-1, Okura Setagaya, Tokyo 157-8535, Japan; 4 Research Center for Medical and Dental Sciences, Tokyo Medical and Dental University, 1-5-45 Yushima, Bunkyo-ku, Tokyo 113-8510, Japan; 5 Department of Molecular and Experimental Medicine, The Scripps Research Institute, 10550 North Torrey Pines Road, MEM-161, La Jolla, California 92037, USA; 6 Department of Orthopaedic Surgery, Surgical Science, Tokai University School of Medicine, 143 Shimokasuya, Isehara, Kanagawa 259-1143, Japan; 7 University of California, San Diego, 9500 Gilman Dr Mail Code 0863, La Jolla, California 92093-0863, USA; 8 CREST from Japan Agency for Medical Research and Development, 1-7-1 Ootemachi, Tiyoda, Tokyo 100-0004, Japan

## Abstract

The main pathogenesis of intervertebral disc (IVD) herniation involves disruption of the annulus fibrosus (AF) caused by ageing or excessive mechanical stress and the resulting prolapse of the nucleus pulposus. Owing to the avascular nature of the IVD and lack of understanding the mechanisms that maintain the IVD, current therapies do not lead to tissue regeneration. Here we show that homeobox protein Mohawk (Mkx) is a key transcription factor that regulates AF development, maintenance and regeneration. Mkx is mainly expressed in the outer AF (OAF) of humans and mice. In *Mkx*^−/−^ mice, the OAF displays a deficiency of multiple tendon/ligament-related genes, a smaller OAF collagen fibril diameter and a more rapid progression of IVD degeneration compared with the wild type. Mesenchymal stem cells overexpressing Mkx promote functional AF regeneration in a mouse AF defect model, with abundant collagen fibril formation. Our results indicate a therapeutic strategy for AF regeneration.

## Abstract (web summary)

Homeobox protein Mohwak (Mkx) is involved in tendon and ligament development. Here the authors show that Mkx in the outer annulus fibrosus of the intervertebral disc plays a role in maintenance of the IVD, showing that stem cells overexpressing Mkx enhance therapeutic IVD regeneration in mice.

## Graphical abstract

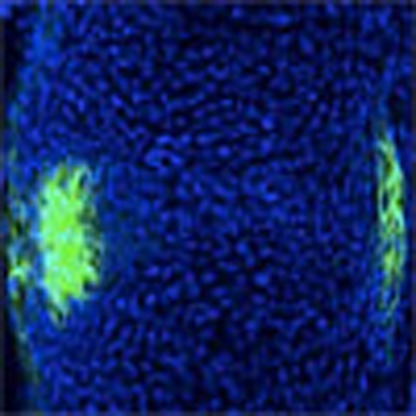

The spine is composed of vertebral bodies connected by intervertebral discs (IVDs) and plays a central role in human movement, as it enables standing, twisting and bending positions. Rupture of the exterior section of the IVD known as the annulus fibrous (AF), especially by degenerative changes in the outer AF (OAF), can cause IVD herniation, which is a common and severe disease associated with pain and disability. Although standard discectomy provides pain relief, this technique reduces the mechanical property of the AF and accelerates progression to IVD degeneration and degenerative spondylosis. To date, tissue regenerative therapy of the IVD has not been achieved, partly because the IVD is the largest avascular tissue and has poor self-healing potential^1^, and because the specific master transcription factors that regulate IVD development and homeostasis are unknown^2^,^3^. A better understanding of the molecular mechanism of IVD development and homeostasis is required for the goal of regenerative therapy of IVD degeneration.

The IVD can be distinguished by its three major components, the nucleus pulposus (NP), the AF and the cartilaginous endplate (EP). The NP is a jelly-like material located in the centre of the disc, and surrounding it is fibrous tissue of the AF. The EP extends superiorly and inferiorly over the inner AF (IAF) and the NP. The AF comprises the OAF and the IAF. The OAF is a highly organized collagenous structure consisting mainly of type I collagen^4^,^5^,^6^. The expansion pressure of the NP and the tensile strength of the AF are balanced and control the mechanical properties of the IVD. The EP, which has specialized capillary beds, provides nutrition to the whole IVD^7^. The developmental origin of these components is different. At E12.0 in the mouse, sclerotome cells migrate and condense around the notochord^8^. These cells form a metameric pattern of more-condensed regions, which become vertebral bodies, and less-condensed regions, which form the AF. The notochord expands within the future IVD, to form the NP^9^. Thus, the molecules that promote disc regeneration are expected to be different between these tissues.

To identify tissue-specific transcription factors present during embryogenesis, we previously generated a whole-mount *in situ* hybridization (ISH) database, EMBRYS, that covers the expression of ∼1,500 transcription factors and cofactors during embryogenesis^10^. Among them, we and others found that homeobox protein Mohawk (Mkx) is specifically expressed in tendon-related and ligament-related tissues, and could be used to promote tendon regeneration^11^,^12^,^13^,^14^,^15^,^16^. Mkx is a member of the three-amino-acid loop (TALE) superclass of atypical homeobox genes belonging to the Iroquois family^17^. The expression of *Mkx* in the syndetome is detectable at E12.5 and its expression is maintained even in matured ligament cells^17^. The ligament-like properties of IVDs, especially the AF, which connect the adjacent upper and lower vertebrae, and contribute biomechanically to the stabilization of the spinal motion segment, prompted us to analyse *Mkx* expression in detail during AF development. By immunohistochemistry (IHC) and ISH, Mkx is mainly expressed in the outer AF (OAF) of humans and mice. In *Mkx*^−/−^ mice, the OAF displays a deficiency of multiple tendon/ligament-related genes, a smaller OAF collagen fibril diameter and a more rapid progression of IVD degeneration with age compared with the wild type. Overexpression of Mkx in mesenchymal stem cells (MSCs) promotes tenocyte-like differentiation *in vitro* and these cells can contribute to functional AF regeneration in a mouse AF defect model with abundant collagen fibril formation *in vivo*. The present work therefore reveals that Mkx is a central transcription factor that regulates AF development, maintenance and regeneration.

## Results

### Mkx is mainly expressed in the OAF of humans and mice

**Figure 1:**
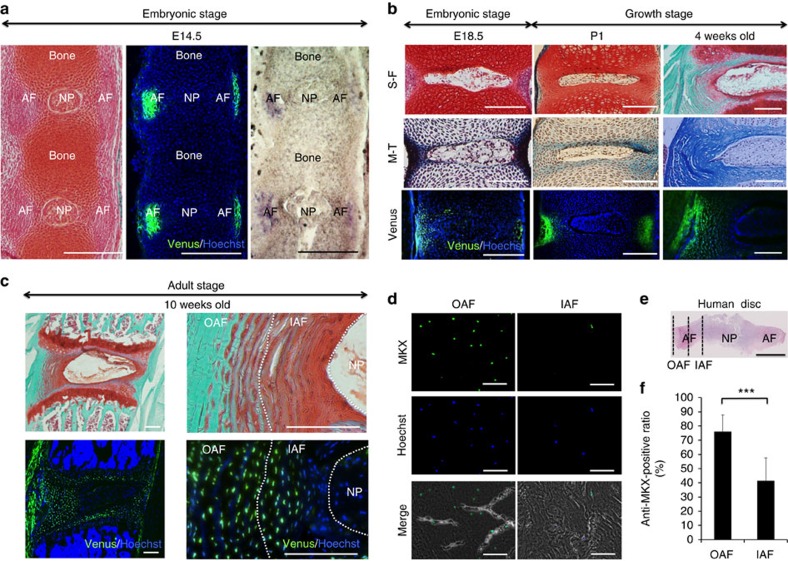
Mohawk gene *(Mkx)* is expressed in the outer annulus fibrosus (OAF). (**a**) Representative images of intervertebral discs (IVDs) of E14.5 mice. Left: Safranin O fast green stain in a wild-type mouse. Middle: IHC of a Venus knock-in mouse. Green: anti-GFP. Blue: Hoechst. Right: *in situ* hybridization of *Mkx* of a Venus knock-in mouse. Scale bars, 200 μm. (**b**) Representative disc images from embryonic stages to growth stages (upper and middle panels, Safranin O fast green (S-F) stain and Masson's trichrome (M-T) stain in a wild-type mouse; lower panel, IHC of a Venus knock-in mouse; green, anti-GFP; blue; Hoechst). Scale bars, 200 μm. (**c**) Representative images of IVDs from 10-week-old mice (upper panel, safranin O fast green stain in a wild-type mouse; lower panel, IHC of a Venus knock-in mouse; green; anti-GFP; blue; Hoechst). Scale bars, 200 μm. (**d**) Immunohistochemistry of the coronal sections of a human lumbar disc IAF and OAF (#1: 20-year-old male, L1/2). Green: anti-MKX. Blue: Hoechst. Scale bars, 100 μm. (**e**) Overview of the observed area of the IAF and OAF. The section is a haematoxylin–eosin staining of a human lumbar disc (#1: 20-year-old male, L1/2). Dotted lines indicate borders of the NP, IAF and OAF. Scale bar, 1 cm. (**f**) Percentages of human IAF and OAF cells that were positive for MKX in each zone. Values are the mean of nine discs from five donors. Error bars represent s.e.m. ***P<0.001. Statistical differences were assessed with Student's *t*-test. GFP, green fluorescent protein; MKX, Mohawk homeobox protein; P1, postnatal day 1.

To investigate the expression of *Mkx* in IVD, we used Mkx-Venus knock-in mice. The details about these mice were previously reported^11^. The endogenous expression of Mkx in the IVD, as determined by ISH, shadowed the Venus expression observed in Mkx-Venus knock-in mice by IHC at E14.5. This suggests that the expression of Mkx-Venus is consistent with the endogenous expression pattern of Mkx in the IVD (Fig. 1a). In IHC, Mkx-Venus was strongly expressed at E14.5 in the OAF of the somite, and its expression was continued even at the later stages (Fig. 1a,b). Importantly, the expression of Venus was specifically localized in the OAF, even after disc development was completed, in 10-week-old mice (disc formation is completed by approximately postnatal week 8 in mice^18^). Conversely, Venus expression in the IAF decreased gradually as it approached the NP region (Fig. 1c). We also evaluated the expression of MKX in human lumbar discs (Fig. 1e (details of regions between the OAF and IAF); Supplementary Table 1). Consistent with the observation in mice, more MKX-positive cells were observed in the OAF compared with the IAF (Fig. 1d,f). These results suggest that *Mkx* is expressed mainly in the OAF.

### Mkx deficiency affects development of AF

**Figure 2:**
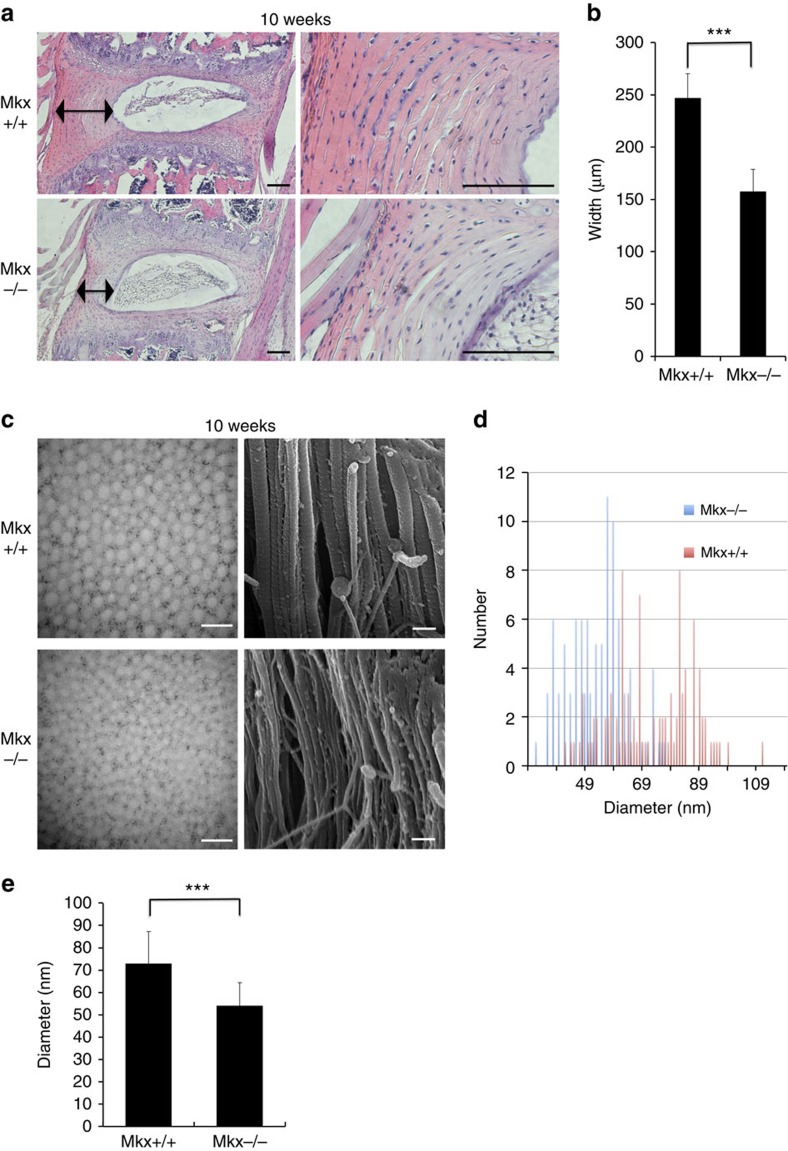
Multiscale analyses of the annulus fibrosus (AF) from *Mkx*^−/−^ mice. (**a**) Haematoxylin–eosin staining of IVDs of 10-week-old *Mkx*^+/+^ and *Mkx*^−/−^ mice. Black arrow, width of the AF. Scale bars, 300 μm. (**b**) Width of the AF from 10-week-old *Mkx*^+/+^ versus *Mkx*^−/−^ mice. For Mkx^+/+^, mean width=246.9 μm (s.d.=23.04 μm). For *Mkx*^−/−^, mean width=157.5 μm (s.d.=21.16 μm). (**c**) Images of transmission electron microscopy (left column) and scanning electron microscopy (right column) of the OAF of 10-week-old *Mkx*^+/+^ and *Mkx*^−/−^ mice. Scale bars, 200 nm. (**d**) Histogram of the diameter of collagen fibrils from 10-week-old *Mkx*^+/+^ and *Mkx*^−/−^ mice. Blue bar, *Mkx*^−/−^; red bar, *Mkx*^+/+^. (**e**) The calculated mean of the diameter of 100 collagen fibrils from 10-week-old *Mkx*^+/+^ and *Mkx*^−/−^ mice. For *Mkx*^+/+^, mean diameter=72.73 nm (s.d.=14.5 nm). For *Mkx*^−/−^, mean diameter=53.99 nm (s.d.=10.31 nm). Error bars represent the s.d. ***P<0.001. Statistical differences were assessed with Student's *t*-test.

**Figure 3:**
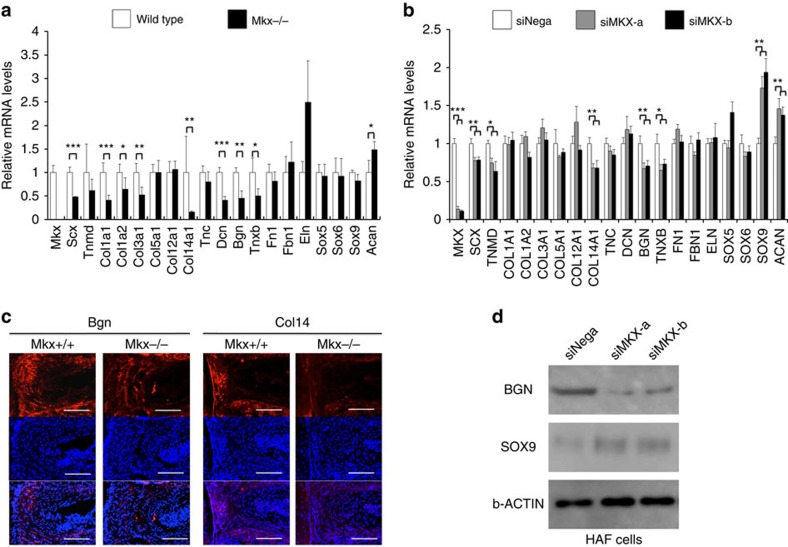
Molecular analyses of the annulus fibrosus (AF) cells. (**a**) Quantitative real-time reverse transcription PCR (qRT–PCR) analyses of the expression of ligament-related genes in *Mkx*^+/+^ and *Mkx*^−/−^ mice. The mRNA levels of ligament markers in *Mkx*^+/+^ mice were normalized to 1. Error bars represent s.e.m. *P<0.05, **P<0.01, ***P<0.001. Statistical differences were assessed with Student's *t*-test. (**b**) qRT–PCR analyses of ligament- and cartilage-related gene expression in human AF cells transfected with a small interfering RNA for *MKX* (si*MKX*-a and si*MKX*-b) or the negative control (siNega). The mRNA levels of ligament markers in human AF (HAF) primary cultured cells transfected with siNega were normalized to 1. Error bars represent s.e.m. *P<0.05, **P<0.01, ***P<0.001. Statistical differences were assessed with Student's *t*-test. (**c**) Immunohistochemistry (IHC) of the intervertebral disc (IVD) of 10-week-old *Mkx*^+/+^ and *Mkx*^−/−^ mice. IHC shows little decreased Bgn and Col14 protein expression in *Mkx*^−/−^ mice compared with *Mkx*^+/+^ mice. Red: Bgn and Col14. Blue: DAPI. Scale bars, 100 μm. (**d**) Western blot analyses of human AF cells. Western blotting showed decreased BGN and increased SOX9 protein expression following siMKX induction of HAF cells.

We examined the function of *Mkx* in the OAF using *Mkx*^+/+^ and *Mkx*^−/−^ mice. In haematoxylin and eosin (HE) staining, AF cell number did not differ between *Mkx*^+/+^ and *Mkx*^*−/−*^ mice at 10 weeks. For *Mkx*^+/+^ mice, the mean cell number was 213. For *Mkx*^−/−^ mice, the mean cell number was 217. The *P* value was 0.51. However, HE staining revealed that AF collagen fibres were thinner in *Mkx*^−/−^ mice than they were in *Mkx*^+/+^ mice (Fig. 2a,b). An electron microscopy-based analysis confirmed that the diameter of collagen fibrils was significantly narrower in *Mkx*^−/−^ mice than in *Mkx*^+/+^ mice at 10 weeks (Fig. 2c–e). This difference in phenotype was also confirmed at postnatal day 1 (P1; Supplementary Fig. 1). We next examined the effect of *Mkx* on gene expression levels using OAF cells isolated from the tail discs (C1/2-8/9) of 10-week-old *Mkx*^+/+^ or *Mkx*^−/−^ mice (Supplementary Fig. 2). Quantitative real-time reverse transcription PCR (qRT–PCR) analyses revealed the downregulation of (i) a set of collagen genes, including *Col1a1* and *Col1a2*, which were the main components of the OAF; (ii) various small leucine-rich proteoglycan (SLRP) family genes (especially *Biglycan* (*Bgn*), which is expressed mainly in the OAF and plays an essential role in the maintenance of OAF homeostasis)^19^,^20^,^21^ and (iii) the tendon-related transcription factor *Scleraxis* (*Scx*)^22^ in *Mkx*^−/−^ compared with *Mkx*^+/+^ cells (Fig. 3a). The *P* value of *Elastin* (*Eln*) was 0.084 and a significant difference between Mkx+/+ mice and Mkx−/− mice at 10 weeks was not seen. Thus, we judged that Mkx was not a definitive contributor to Eln expression. In IHC, Bgn and Col14 protein expression were decreased in *Mkx*^−/−^ mice compared with *Mkx*^+/+^ mice at 10 weeks (Fig. 3c). We also investigated the knockdown effect of *MKX* in human AF (HAF) primary cultured cells and found that the expression of tendon/ligament-related genes, such as those encoding *SCX*, *TNMD*, *COL14A1*, *TNC*, *BGN* and *TNXB*, was significantly downregulated, whereas expression of the cartilage-related genes *SOX9* and *ACAN* was upregulated (Fig. 3b). Western blot analysis confirmed the downregulation of BGN and upregulation of SOX9 following MKX knockdown in HAF cells (Fig. 3d). Taken together, in mice and human, *Mkx* has an important role in the expression of some collagen and SLRP genes in the OAF. These results also indicate a role for *Mkx* in collagen fibril formation during development, including adulthood.

**Figure 4:**
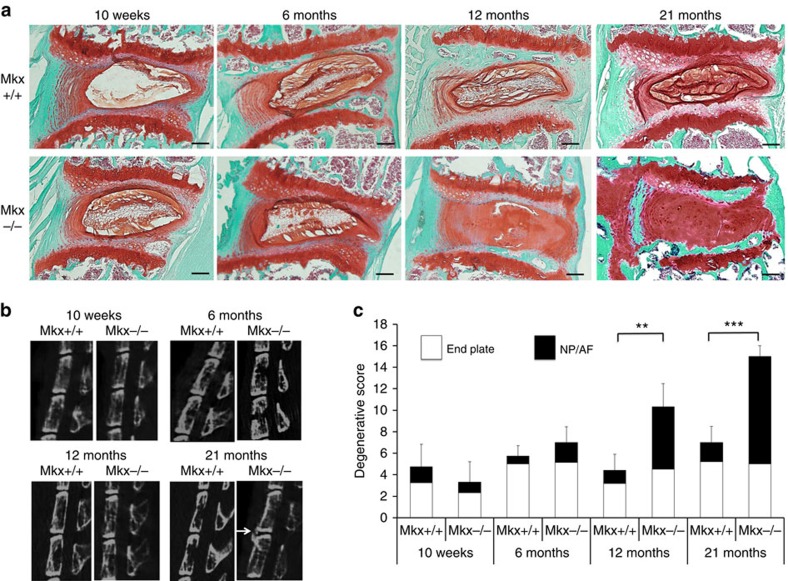
*Mkx*^−/−^ mice develop early onset disc degeneration. (**a**,**b**) Histological and computerized tomography (CT) changes with age between *Mkx*^+/+^ and *Mkx*^−/−^ mice. Safranin O fast green staining of sagittal sections of L3/4 (**a**) and sagittal CT images of the lumbar spine (**b**) at 10 weeks, and 6, 12 and 21 months. Scale bars, 300 μm. (**c**) Histological degenerative scores of intervertebral discs (IVDs) in *Mkx*^+/+^ and *Mkx*^−/−^ mice. Error bars represent s.e.m. **P<0.01, ***P<0.001. Statistical differences were assessed with Student's *t*-test.

Next, to test whether the *Mkx*^−/−^ phenotype has an effect on the IVD degeneration that occurs during the aging process, we analysed *Mkx*^+/+^ and *Mkx*^−/−^ mice using HE staining and Safranin O fast green staining at various stages. The OAF collagen fibres in *Mkx*^−/−^ mice were thinner than those in *Mkx*^+/+^ mice at all ages (Supplementary Fig. 3a). In terms of IVD degeneration, there were no clear differences between *Mkx*^+/+^ and *Mkx*^−/−^ mice at 10 weeks and 6 months; however, at 12 and 21 months, *Mkx*^−/−^ mice showed high levels of degenerative changes in lumbar discs, whereas the lumbar discs of *Mkx*^+/+^ mice did not show changes from those observed at 10 weeks. At 12 and 21 months, small round cells, morphologically resembling chondrocytes, were observed in the NP and IAF of *Mkx*^−/−^ mice (Fig. 4a; Supplementary Fig. 3b). The NP and the IAF were stained more strongly with Safranin O, indicating degenerative changes. To further analyse IVD in *Mkx*^−/−^ mice, we performed IHC for Venus, Col1, CD24 and KRT18 using 21-month-old *Mkx*^+/+^ mice and *Mkx*^−/−^ mice. Col1 is a main component of the AF and Venus shadows Mkx expression. CD24 and KRT18 are well characterized as NP cell markers^23^,^24^,^25^,^26^,^27^. As expected, Venus expression is restricted at AF of 21-month-old *Mkx*^−/−^mice. Col1 expression was observed in the AF of both 21-month-old *Mkx*^*+/+*^ and *Mkx*^−/−^ mice, but not in the NP region of *Mkx*^*+/+*^ and *Mkx*^−/−^ mice (Supplementary Fig. 4a). KRT18 was observed in the NP region of both 21-month-old *Mkx*^*+/+*^ and *Mkx*^−/−^ mice. On the contrary, CD24-positive cells were observed only in the NP of 21-month-old *Mkx*^*+/+*^ mice but not in *Mkx*^−/−^ mice (Supplementary Fig. 4b). These results suggested that the cells at NP region of 21-month-old *Mkx*^−/−^ mice do not possess characters of neither normal AF cells nor normal NP cells.

In microcomputerized tomography views of the lumbar spine, the disc height index between L6 and S1 was measured in *Mkx*^*+/+*^ and *Mkx*^−/−^ mice^28^. As a result, there was no significant difference between the two groups at 10 weeks, but disc height index was clearly reduced in *Mkx*^−/−^ mice at 21 months (Supplementary Fig. 5a–c). Moreover, at 21 months, bone spur formations were observed in the lumbar spine of *Mkx*^−/−^ mice (Fig. 4b; Supplementary Fig. 5d). This phenotype could be related to the instability of vertebrae in *Mkx*^−/−^ mice^29^.

The degenerative histological score of *Mkx*^−/−^ mice was higher than that of *Mkx*^+/+^ mice at 12 and 21 months (Fig. 4c)^30^. These degenerative changes were also observed in the cervical–thoracic and sacral–caudal junctions in *Mkx*^−/−^ mice. It was predicted that the mechanical stress on IVD was related to the degenerative change of IVD of *Mkx*^−/−^ mice because the changes did not occur in all IVDs. These results indicate the essential role of *Mkx* in collagen fibril formation in the OAF during developmental stages, and its function as a stabilizer in IVDs.

### The ability of Mkx for the differentiation of MSC

**Figure 5:**
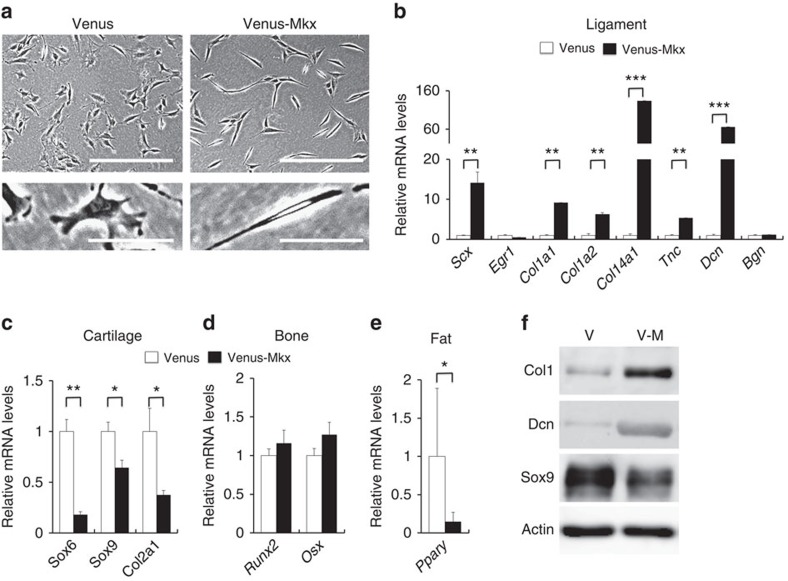
*Mkx* promotes the differentiation of mesenchymal stem cells to ligament cells. (**a**) Images of C3H10T1/2 cells induced by Venus (C3H10T1/2-V or Venus) and Venus-Mkx (C3H10T1/2-VM or Venus-Mkx) in plate culture. Scale bars, 500 μm (top); 100 μm (bottom). (**b**–**e**) Quantitative real-time reverse transcription PCR (qRT–PCR) analyses of the expression of ligament, cartilage, bone and fat-related genes in C3H10T1/2-V and C3H10T1/2-VM cells. mRNA levels in C3H10T1/2-V cells were normalized to 1. Error bars represent s.e.m. *P<0.05, **P<0.01, ***P<0.001. Statistical differences were assessed with Student's *t*-test. (**f**) Western blot analyses of C3H10T1/2-V and C3H10T1/2-VM cells for lineage markers.

Cell transplantation has been proposed as an effective strategy to treat IVD degeneration. Among the various cell sources available, MSCs either from the bone marrow or adipose tissue have been proved to facilitate IVD repair in animal models^31^. To test whether MSCs with *Mkx* induction are a therapeutic option for IVD regeneration, we overexpressed Venus-Mkx in C3H10T1/2 cells using retroviruses (C3H10T1/2 cells induced by Venus-Mkx are referred to as C3H10T1/2-VM or Venus-Mkx, and C3H10T1/2 cells induced only by Venus are referred to as C3H10T1/2-V or Venus). Venus-Mkx overexpression markedly induced the differentiation of C3H10T1/2 cells into a spindle-shaped cell type, with the upregulation of multiple ligament-related genes, such as those encoding *Scx*, *Col1a1*, *Col1a2*, *Col14a1*, *Tnc* and *Dcn* (Fig. 5a,b). The downregulation of cartilage- and fat-related genes was also observed (Fig. 5c,e), whereas the expressions of bone-related genes were not much changed (Fig. 5d). Western blot analyses of Col1, Dcn and Sox9 were consistent with the results of the qRT–PCR (Fig. 5f). We also confirmed the above data observed with C3H10T1/2 using mouse bone marrow-derived MSCs (Supplementary Fig. 6). These data indicate that *Mkx* promotes differentiation of MSCs to tendon/ligament-like cells in gene expression levels, consistent with the previous reports^14^,^15^.

**Figure 6:**
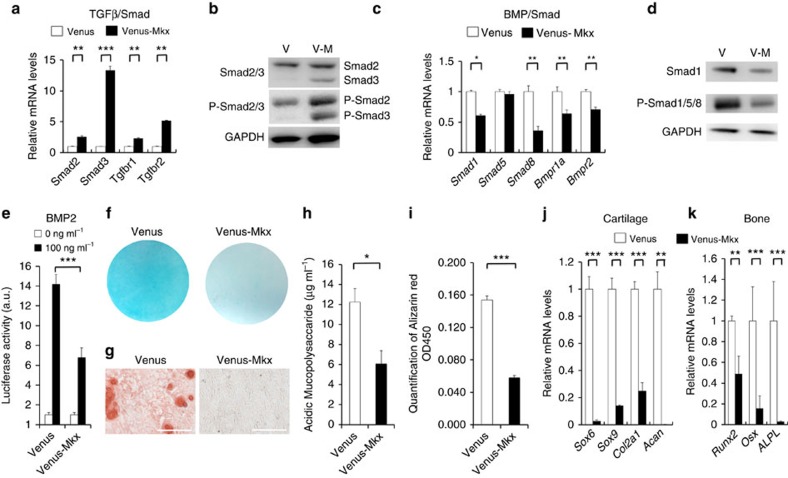
Link between *Mkx* and TGFβ and BMP signalling pathway component during ligament differentiation. (**a**,**c**) qRT–PCR analyses of the expression of TGFβ/Smad- and BMP/Smad-related genes in C3H10T1/2-V and C3H10T1/2-VM cells. mRNA levels in C3H10T1/2-V cells were normalized to 1. Error bars represent s.e.m. *P<0.05, **P<0.01, ***P<0.001. Statistical differences were assessed with Student's *t*-test. (**b**,**d**) Western blot analyses of C3H10T1/2-V and C3H10T1/2-VM cells. TGFβ-dependent phosphorylation of Smads is shown by anti-p-Smad2/3 antibodies. BMP-dependent phosphorylation of Smads is shown by anti-p-Smad1/5/8 antibodies. (**e**) Luciferase assay to determine responsiveness to BMP2. Id1-Bre luciferase was transfected into C3H10T1/-V and C3H10T1/2-VM cells, followed by treatment with or without BMP2 (100 ng ml^−1^). Fold-induction values in the presence of BMP2 compared with the basal levels of each cell line are indicated. **P<0.01. Statistical differences were assessed with Student's *t*-test. (**f**–**k**) C3H10T1/2-V and C3H10T1/2-VM cells were subjected to chondrocyte (**f**,**h**,**j**) and osteocyte (**g**,**i**,**k**) differentiation. (**f**) Alcian blue staining of C3H10T1/2-V and C3H10T1/2-VM cells after induction of chondrocyte differentiation. (**g**) Alizarin red staining of C3H10T1/2-V and C3H10T1/2-VM cells after induction of osteocyte differentiation. Scale bars, 200 μm. (**h**) Mucopolysaccaride quantification after chondrogenic induction. Error bars represent s.e.m. *P<0.05. Statistical differences were assessed with Student's *t*-test. (**i**) Calcification evaluation after osteogenic induction. Error bars represent s.e.m. ***P<0.001. Statistical differences were assessed with Student's *t*-test. (**j**,**k**) qRT–PCR analyses of mesodermal gene expression in C3H10T1/2-V and C3H10T1/2-VM cells in the presence or absence of induction with BMP2. mRNA levels in C3H10T1/2-V cells without induction were normalized to 1. Error bars represent s.e.m. **P<0.01, ***P<0.001. Statistical differences were assessed with Student's *t*-test. BMP, bone morphogenetic protein; TGFβ, transforming growth factor β.

To gain insight into the molecular mechanisms underlying the determination of cell lineage by *Mkx*, we performed microarray analyses of these C3H10T1/2 cells. In addition to the dynamic upregulation of multiple tendon/ligament-related markers and downregulation of cartilage-related markers, transforming growth factor β (TGFβ)-related genes were upregulated and bone morphogenetic protein (BMP)-related genes were downregulated in C3H10T1/2 cells (Supplementary Fig. 7a,b). Previous reports revealed that TGFβ functions in IVDs to promote the differentiation of AF from the sclerotome and to prevent chondrocyte differentiation in the presumptive IVD^32^. Other reports suggested that disc cells continue to respond to TGFβ signalling during postnatal growth^33^ and that BMP signalling was related to IVD degeneration^34^. However, the role of *Mkx* in these pathways remains unclear. Therefore, further analyses were performed. qRT–PCR revealed that *Smad2*, *Smad3*, *TGFbr1* and *TGFbr2* were upregulated (Fig. 6a), whereas *Smad1*, *Bmpr1a* and *Bmpr2* were downregulated (Fig. 6c). Western blot analysis also showed that Smad3 and p-Smad2/3 were increased, whereas Smad1 and p-Smad1/5/8 were decreased (Fig. 6b,d). Luciferase assay using BMP-responsive luciferase reporter revealed that C3H10T1/2-VM exhibited reduced luciferase activity with BMP2 stimulation compared with C3H10T1/2-V (Fig. 6e)^35^. These results suggest that changes in the *Mkx*-dependent *Smads* ratio in C3H10T1/2 account for the promoted signal responses to TGFβ and the reduced responsiveness to BMP signals. Furthermore, these changes in signal responsiveness may partly explain the promotive function in the IVD, the ligament differentiation of MSCs^36^,^37^ and the suppression of differentiation into other lineages and IVD degeneration^22^. So we examined the mesodermal-lineage induction in C3H10T1/2-V and C3H10T1/2-VM using BMP2. In C3H10T1/2-VM cells, the induction of differentiation into chondrocytes and osteocytes was severely disrupted (Fig. 6f–i). qRT–PCR analyses showed either very little or no expression in multiple cartilage- and bone-related markers compared with C3H10T1/2-V cells (Fig. 6j,k). Adipogenic differentiation was also inhibited by Mkx overexpression (Supplementary Fig. 8). These results suggest that Mkx act positively on the property maintenance of the AF cells and suggest that MSCs overexpressing *Mkx* can be effective as transplanted cells.

### The possibility of Mkx for therapeutic tools of OAF

**Figure 7:**
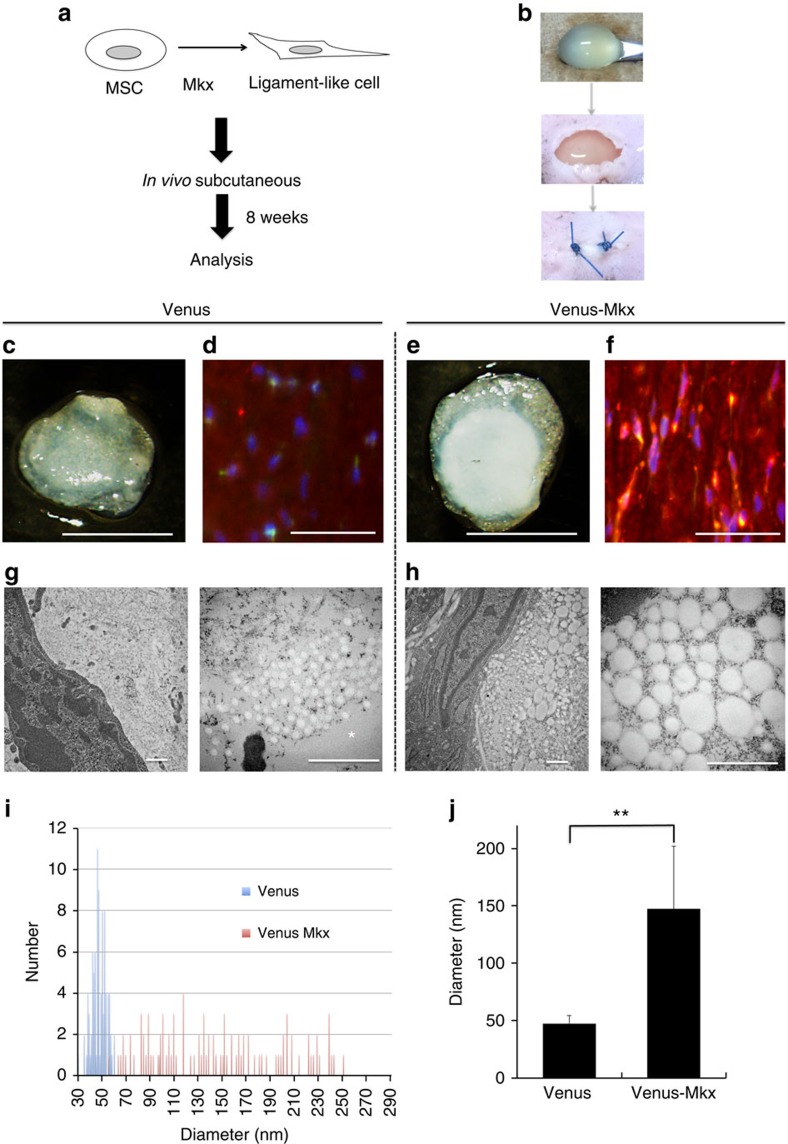
*Mkx*-induced mesenchymal stem cells form tissue consisting of type I collagen. (**a**,**b**) Strategy for testing the type I collagen synthesis ability of C3H10T1/2 cells induced by Venus (C3H10T1/2-V or Venus) and Venus-Mkx (C3H10T1/2-VM or Venus-Mkx) after subcutaneous transplantation. (**c**,**e**) Gross appearance of products 8 weeks after subcutaneous transplantation (**c**: C3H10T1/2-V; **e**: C3H10T1/2-VM). Scale bars, 1 mm. (**d**,**f**) IHC of these products (**d**: C3H10T1/2-V; **f**; C3H10T1/2-VM). Blue, Hoechst; green, anti-GFP; red, anti-Col1a1. Scale bars, 50 μm. (**g**,**h**) Images of transmission electron microscopy (**g**: C3H10T1/2-V; **h**: C3H10T1/2-VM). * The area of the collagen gel. Scale bars, 500 nm. (**i**) Histogram of the diameter of collagen fibrils from C3H10T1/2-V and C3H10T1/2-VM cells. Blue bar, C3H10T1/2-V; red bar, C3H10T1/2-VM. (**j**) The calculated mean of the diameter of 100 collagen fibrils from C3H10T1/2-V and C3H10T1/2-VM cells. The mean diameter of 100 collagen fibrils was calculated: for C3H10T1/2-V cells, mean diameter=46.99 nm (s.d.=7.409 nm); for C3H10T1/2-VM cells, mean diameter=147.32 nm (s.d.=54.54 nm). Error bars represent the s.d. **P<0.01. Statistical differences were assessed with Student's *t*-test. GFP, green fluorescent protein.

To test whether MSCs overexpressing *Mkx* are capable of producing collagen fibres, which can be used for OAF regeneration *in vivo*, we first performed an *in vitro* three-dimensional culture experiment using a chemical gel without animal collagen. After 8 weeks, we performed IHC for Col1a1 and found that C3H10T1/2-VM cells had a higher capacity for type I collagen synthesis compared with C3H10T1/2-V cells (Supplementary Fig. 9). On the basis of these results, we next examined whether C3H10T1/2-VM cells inserted in a type I collagen scaffold, with greater rigidity than a chemical gel, embedded subcutaneously in dorsal skin pockets of mice had the potential to form collagen fibrils (Fig. 7a–c,e). The control group was set to the group of C3H10T1/2-V cells. After 8 weeks, the gels were excised and evaluated. Histologically, thicker tissues were generated in the C3H10T1/2-VM cell group than in the control group (Supplementary Fig. 10). No mesodermal tissues such as cartilage, bone, or fat were observed in either group. IHC revealed abundant type I collagen synthesis in the C3H10T1/2-VM scaffold (Fig. 7d,f).

Transmission electron microscopy (TEM) revealed that the diameter of collagen fibrils of the C3H10T1/2-VM scaffold was clearly larger compared with that of the C3H10T1/2-V scaffold (Fig. 7g–j). TEM also revealed that collagen fibrils were hardly confirmed in the control group (that is, only transplanted gel was observed) compared with the C3H10T1/2-V group (Supplementary Fig. 11). Although we could not distinguish whether the collagen in the fibril is newly synthesized by the cells or originates from the gel, but the amount of collagen increase and the highly organized structure suggest that at least some of the type I collagen observed newly synthesized as a result of *Mkx* overexpression. These results demonstrated that MSCs overexpressing *Mkx* had a strong ability not only to express collagens and proteoglycans, but also to synthesize collagen fibrils even in an environment different from the OAF.

**Figure 8:**
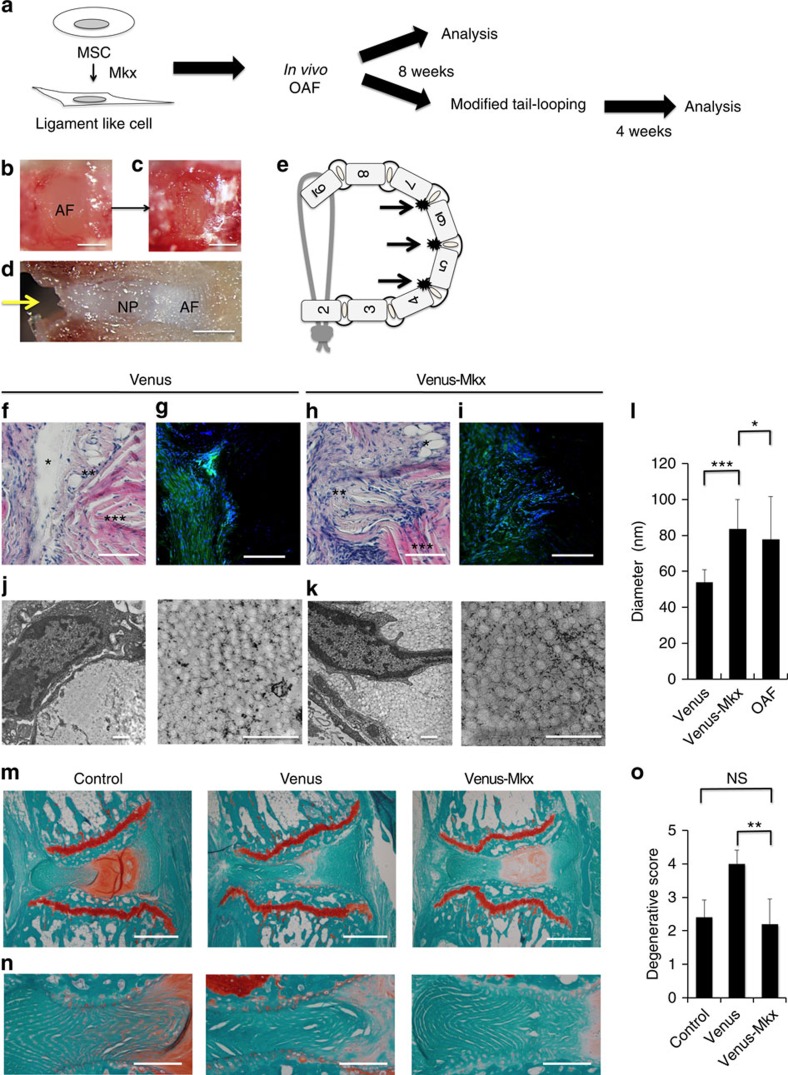
*Mkx*-induced mesenchymal stem cells promote functional AF regeneration. (**a**) Strategy for assessing type I collagen synthesis of C3H10T1/2 cells induced by Venus (C3H10T1/2-V or Venus) and Venus-Mkx (C3H10T1/2-VM or Venus-Mkx) after outer AF (OAF) transplantation. (**b**) OAF before creating a transplantation site. Scale bar, 200 μm. (**c**) OAF after creating a transplantation site. Scale bar, 200 μm. (**d**) Cut surface of intervertebral disc (IVD) after creating a transplantation site. Yellow arrow, transplantation site. Scale bar, 200 μm. (**e**) Schema of the modified tail-looping model. Black arrow, transplanted site. (**f**,**h**) Haematoxylin and eosin staining of the transplantation sites of the OAF (**f**: C3H10T1/2-V; **h**: C3H10T1/2-VM). * The area in which the collagen gel was dissolved. ** Newly synthesized tissue. *** OAF. Scale bars, 50 μm. (**g**,**i**) IHC of these products (**g**: C3H10T1/2-V; **i**: C3H10T1/2-VM). Blue, Hoechst; green, anti-GFP. Scale bars, 50 μm. (**j**,**k**) Images of transmission electron microscopy (**j**: C3H10T1/2-V; **k**: C3H10T1/2-VM). Scale bars, 500 nm. (**l**) The calculated mean of the diameter of 100 collagen fibrils from C3H10T1/2-V and C3H10T1/2-VM cells. The mean diameter of 100 collagen fibrils was calculated: for C3H10T1/2-V cells, mean diameter=53.72 nm (s.d.=7.281 nm); for C3H10T1/2-VM cells, mean diameter=83.54 nm (s.d.=16.34 nm); for the intact OAF of C3H/HeSlc mice, mean diameter=77.74 nm (s.d.=24 nm). Error bars represent the s.d. **P<0.01. (**m**,**n**) Representative images of IVDs in each group. Safranin O fast green staining. (**m**) Whole images of each group. Transplantation sites of C3H10T1/2-V or C3H10T1/2-VM cells are on the left side in the images. Scale bars, 300 μm. (**n**) Images with a focus on the transplanted side of the AF. Scale bars, 100 μm. (**o**) Histological grading score of the AF using the Nishimura system. The mean average grade: control=2.4 (s.d.=0.5164); Venus=4 (s.d.=0.4082); Venus-Mkx=2.2 (s.d.=0.7528). **P<0.01. Statistical differences were assessed with Student's *t*-test. GFP, green fluorescent protein.

Next, we investigated whether MSCs overexpressing *Mkx* have the capacity to repair AF defects. A solution of liquefied collagen gel containing C3H10T1/2-V or C3H10T1/2-VM cells was placed in the cavity of the AF (Fig. 8a–d). After 8 weeks, the discs were excised and evaluated. HE staining and IHC revealed that, although the reconstruction of the structure of lamella was not confirmed, abundant tissue was synthesized in the OAF transplanted with C3H10T1/2-VM cells (Fig. 8f–i). TEM analysis revealed that there were also abundant collagen fibrils in the tissues that were transplanted with C3H10T1/2-VM cells in the entire field, even at low magnification; in contrast, there were few collagen fibrils in the tissues that were transplanted with C3H10T1/2-V cells (Fig. 8j,k). In addition, the diameter of the collagen fibrils of C3H10T1/2-VM tissues was larger than that of C3H10T1/2-V tissues (Fig. 8l). Moreover, the diameter of these fibres was almost equal in size to those of intact OAF of C3H/HeSlc mice (Fig. 8l). Although the detailed mechanism underlying these observations remains unknown, these results suggest that the environment around the transplant contributes to the regulation of collagen fibril formation. Subsequently, to examine whether the newly formed tissue had sufficient physical property against mechanical stress and could inhibit IVD degeneration, we performed a modified tail-looping model, in which the uneven mechanical loading of the IVD was applied to the transplanted site in this experiment^38^ (Fig. 8e) and compared the degenerative changes of the whole AF. Wild-type C3H/HeSlc mice with tail-looping were used as a control group. After 4 weeks, the discs were excised and evaluated. In Safranin O fast green staining, the AF of C3H10T1/2-V group was severely disrupted and the red area, which stained aggrecan in the NP, was reduced (Fig. 8m,n). In contrast, the AF of C3H10T1/2-VM group was only mildly disrupted compared with the control group and NP also kept its structure although the red stain of Safranin O was reduced (Fig. 8m). The histological grading of the AF by Nishimura system showed that the control group and the C3H10T1/2-VM group were not significantly different and it was significantly higher in the C3H10T1/2-V group (Fig. 8o)^39^. These results indicate the newly synthesized tissue of C3H10T1/2-VM cells has nearly equal physical property of an intact AF against compression force.

## Discussion

The biomechanical properties of the IVD are produced by the structure in which AF surrounds the NP to provide enough flexibility and alleviate the pressure^6^,^40^. Extensive mechanical stress or aging could be a cause of IVD degeneration including disc herniation with serious clinical symptoms, such as lower back pain and leg pain, which restricts the patients' quality of life. To prevent degeneration of IVD and to develop regenerative therapies for IVD, it is necessary to understand the molecular program of IVD development and homeostasis. The developmental molecular mechanisms of NP and IAF have recently been revealed to some extent. *Sonic Hedgehog (Shh)* and transcription factors *Fox1a* and *Fox2a* are essential for the formation of the NP in mouse embryos^41^,^42^. *Sox5*, *6* and *9* also play important roles in the formation and maintenance of NP and IAF^43^,^44^. There are also some reports regarding the maintenance of NP: the basic fibroblast growth factor was required for the maintenance of the properties of NP cells^45^, and inhibition of tumour necrosis factor α production positively enhanced NP tissue growth^46^.

In contrast, there has been very limited information about the molecular network regulating OAF development. Here we reveal that *Mkx* was expressed specifically in the AF, and mainly in the OAF from embryonic stages throughout life (Fig. 1a–c). In addition, we observed severe degenerative phenotype of the AF in *Mkx*^−/−^ mice. *In vitro* analyses in both mouse and human AF cells revealed that *Mkx* regulates the expression of some collagen and SLRPs genes (Fig. 3a,b), the main components of OAF tissues^19^,^20^,^21^,^47^. *Scx* expression was downregulated in *Mkx^−/−^* mice (Fig. 3) and was promoted by *Mkx* overexpression in C3H10T1/2 cells and bone marrow-derived MSCs (Fig. 5a,b; Supplementary Fig. 6). Scx expression (E12.5) precedes Mkx expression (E13.5) during embryogenesis, suggesting that Mkx is not critical for initiating Scx expression; however, Mkx may support continued expression of *Scx* in tendon/ligament and IVD development^48^. In this regard, although the development of the IVD in *Scx*^−/−^ mice at E18.5 is grossly normal^49^, phenotype in intervertebral discs of *Mkx^−/−^* mice and Mkx ability to regenerate IVD could partly be explained by *Scx* regulation^15^,^48^.

Several gene expression patterns are not in concordance between human and mouse AF cells in these experiments (Fig. 3). The discrepancy between these two systems can be explained as follows. First, we investigated the function of Mkx in the process of differentiation from precursor cells to AF cells in the mouse, whereas in human experiments, we investigated the role of MKX in mature AF cells. Second, the effect of siMKX in the human experiment is transient while, in mouse experiments, *Mkx* is genetically deleted.

Egr1 is also an important transcription factor to induce type I collagen tissue^50^. However, Egr1 did not induce Mkx^50^ expression and our experiments revealed that Mkx did not induce Egr1 expression (Fig. 5b). These findings indicate that that the two transcription factors induce type I collagen formation via different pathways. Whether Egr1 is an important factor in the AF is still unknown, and needs to be considered in future research.

Our data also indicated that Mkx is expressed in adult HAF cells, suggesting a potential role for Mkx in adult IVD tissues (Fig. 3b,d). Although the MKX expression in HAF cells during the aging process is unknown so far, we recently reported that MKX expression was decreased with age in the human anterior cruciate ligament^13^. It is of interest to analyse Mkx expression in AF cells during aging or disease.

The function of AF as spacer and cushion between spines can be established by its multilayer composition. Ligament-like tissues are dominant in the OAF, whereas cartilage-like tissues are more abundant in the IAF^6^,^40^,^51^. The gradual transition is shadowing the differences between expressional levels of *Mkx* and *Sox9* in AF. In *Mkx*^−/−^ mice, this transition between IAF and OAF layers are disturbed and the expression of *Sox9* was upregulated in *Mkx* knockdown OAF cells (Fig. 3b). In addition, *Mkx* overexpression in C3H10T1/2 cells downregulated the expression of *Sox9* (Fig. 5c,f). Consistent with this observation, it is reported that *Sox9* expression is downregulated in OAF cells during the differentiation of OAF cells from the *Scx*^+^/*Sox9*^+^ progenitor cells^44^,^52^. These results suggest that multilayer composition between OAF and IAF might be partly regulated by reciprocal regulation between *Mkx* and *Sox9*.

Furthermore, *Mkx*-induced C3H10T1/2 acquired greater responsiveness to TGFβ, but reduced responsiveness to BMP (Fig. 6a–e). As TGFβ has a function not only to promote the differentiation of AF from the sclerotome but also to prevent chondrocyte differentiation in the presumptive IVD^32^,^53^, this shift in signalling responsiveness triggered by *Mkx* may be among the underlying molecular mechanisms maintaining *Mkx*-dependent AF homeostasis.

The collapse of either AF or NP may cause the tissue degeneration of the whole IVD. The collapse of NP and loss of NP property will cause a decrease of mechanical resistance of the AF, followed by the degeneration of the entire IVD^30^,^40^,^54^,^55^. To regain normal NP property, introduction of several growth factors or MSCss into NP has been reported in animal models and in clinical trials^56^,^57^,^58^,^59^,^60^.

Conversely, in this report, after the initial degradation of AF in *Mkx*^−/−^ mice, we also observed the following degenerative changes of the NP tissue, where *Mkx* is not expressed (Fig. 1c). This phenotype may be explained as secondary changes due to insufficient mechanical property of the AF, as previous reports^19^,^21^. The abolishment of critical matrix molecules in the AF of mice, such as *BGN*^19^,^21^ and secreted protein acidic rich in cysteine (*SPARC*)^61^, have been shown to cause the whole IVD degeneration. In these knockout mice, including *Mkx*^−/−^ mice, morphologically resembling chondrocytes were seen in the degenerative NP, the origin of which is unclear. Further cell lineage analysis, such as *Shh* analysis for notochord lineage^41^,^62^,^63^, is required to understand the phenotype in detail.

Regarding the regeneration of damaged OAF, the transplantation of OAF cells or MSCs into injury sites of IVD in animal models have been reported^64^,^65^,^66^. However, the results of these trials have not been satisfactory at some points. The transplanted OAF cells could not maintain its property as OAF cells and were unable to synthesize sufficient collagen fibres^64^. As for the transplantation of MSCs, the differentiation from MSC to OAF cells is not well directed, resulting in the lack of sufficient collagen fibre synthesis^65^,^66^. In an attempt to overcome these issues, we applied MSCs expressing *Mkx*, and successfully directed the MSCs to differentiate into OAF-like cells. These *Mkx*-induced C3H10T1/2 cells acquired the ability to produce enough number and size of collagen fibrils and subsequently formed collagenous tissues. Consistent with this finding, we previously observed that MKX overexpression in human bone marrow MSCs induced multiple ligament-related genes including *SCX* and *COL1A1*, and *SLRPs*^14^. Although the overexpression of *SCX* in human bone marrow MSCs induced only *COL1A1* and *TNMD*^14^, examination of the combination of *MKX* and *SCX* in the formation OAF cells may be worth trying.

Although the lamellar structure of OAF was not fully reconstituted, the regenerated OAF tissues by transplanted *Mkx*-induced C3H10T1/2 cells had sufficient physical biomechanical property, which was tested and confirmed by the tail-looping model experiment (Fig. 8m–o).

These results support the potential application of *Mkx*-induced human MSCs transplantation in human disc degenerative diseases, including disc herniation, and may also improve the current OAF regeneration trial by simple MSC introduction^60^,^67^. More detailed mechanical evaluation of the intervertebral disc should be performed, using a larger animal model, to test resistance to twisting motion^68^.

The regulatory mechanisms of specific and consistent expression of *Mkx* in OAF cells remain unclear. Identification of the enhancer region and its regulatory molecules may provide us with more insight for the application of OAF regeneration therapy via *Mkx* expression.

## Methods

### Animals

Venus knock-in *Mkx* heterozygous mutant mice in a C57BL/6N background carried an insertion of a Venus cassette that inactivates the *Mkx* gene. We inactivated the *Mkx* gene by homologous recombination in embryonic stem cells using a targeting vector to replace the *Mkx* gene from the translation start site to the end of exon 2 with the Venus gene and PGK-neomycin-resistance (PGKneo) cassette^11^. Male C57BL/6N and C3H/HeSlc mice were purchased from the Sankyo Laboratory (Tokyo, Japan). This animal study was approved by the Tokyo Medical and Dental University ethical committee.

### Human samples

The human samples (nine discs from five human donors; Supplementary Table 1) were obtained from the organ banks through the commercial source (Cosmo Bio USA, Carlsbad, CA). The informed consent was obtained from families of donors through the organ bank.

### Cell culture

C3H10T1/2 cells were grown in α-MEM with 10% fetal bovine serum (FBS) and 1% penicillin–streptomycin (PS; all Sigma-Aldrich). PLAT-E cells were grown in DMEM plus 10% FBS (all Sigma-Aldrich). HAF cells (Articular Engineering) were grown in DMEM: Nutrient Mixture F-12 HAM (DMEM:F12HAM) plus 20% FBS, 1% PS and 20 μg ml^−1^ of ascorbate. Mouse OAF cells were isolated as follows. Mkx^−/−^ or C57BL/6N mice were killed by cervical dislocation and tail discs were dissected (T1/2-7/8, 10 mice per group). Using a clean bench, the discs were trimmed and pieces of OAF tissues were obtained. These tissues were incubated with Triple Express (Gibco) for 30 min and 0.25% Liberase (Roche) for 75 min (Supplementary Fig. 2a–c). Isolated cells were grown in α-MEM with 20% FBS and 1% PS (Invitrogen) until day 5, at which point the medium was changed to α-MEM plus 10% FBS and 1% PS (Invitrogen). Cells were retrieved in passage 1 and processed for qRT–PCR analysis. The purity of AF cells was confirmed by examining the number of Venus-positive cells in the isolated *Mkx*^−/−^cells (Supplementary Fig. 2d). All cells were cultured at 37 °C in 5% CO_2_.

### Three-dimensional culture

To perform the three-dimensional cell culture, cells were trypsinized and suspended in α-MEM and then mixed with PanaceaGel (Menicon Life Science) solution at a volume ratio of 1:2. The final concentration of the cells in the hydrogel was 2 × 10^6^ cells per ml. The cells were grown in α-MEM plus 10% FBS and 1% PS (Invitrogen), and incubated in 5% CO_2_ at 37 °C. The medium was replaced with fresh medium every 2–3 days. The gels containing cells were subsequently used for IHC.

### Knockdown of *MKX* in HAF cells

Fifty picomoles of small interfering RNA for *MKX* (Silencer Select, s49084 and s49085, Thermo Fisher) or negative control (Silencer Select Negative Control No.1, 4390843, Thermo Fisher) was transfected into HAF cells (8 × 10^4^ cells per well) using 8 μl of Lipofectamine RNAiMAX (Invitrogen) in six-well plates. After 72 h, total RNAs and cell lysates were extracted and used for qRT–PCR and western blotting, respectively.

### Retrovirus infection

Venus or Venus-Mkx coding sequences were inserted into the pMIGR vector (Addgene). The pMIGR-Venus construct (as a control) or the pMIGR-Venus-Mkx construct was transfected into PLAT-E cells using FugeneHD (Promega). Forty-eight hours later, media were collected, filtered and transferred to the C3H10T1/2 stable cell line in a medium containing puromycin (5 μg ml^−1^). The C3H10T1/2 cells induced with Venus-Mkx are referred to as C3H10T1/2-VM (or Venus-Mkx), and the C3H10T1/2 cells induced only with Venus are referred to as C3H10T1/2-V (or Venus). No clear differences were observed between C3H10T1/2 and C3H10T1/2-V cells in terms of morphology and gene expression. Therefore, C3H10T1/2-V cells were used as control cells.

### Two mouse models for transplantation

C3H/HeSlc mice (male, 10 weeks of age, *n*=3 per group) were anaesthetized with pentobarbital sodium (Somunopentyl; Kyoritsu Seiyaku). Dorsal skin pockets were made and type I collagen gels (Cellmatrix; Kurabo) containing C3H10T1/2-V or C3H10T1/2-VM cells were embedded. The gels containing the cells were prepared in advance, as follows. Trypsinized cells were resuspended in a collagen solution at a concentration of 4 × 10^7^ cells per ml, and 60 μl per well of this solution was placed in a 96-well plate (Cellstar; Greiner Bio-One). After incubation at 37 °C in 5%CO_2_ and gelation, the gels were embedded in the dorsal skin pockets and the skin was sutured. After 8 weeks, the mice were killed by cervical dislocation and the gels were excised. The gels were processed for IHC and TEM.

C3H/HeSlc mice (male, 10 weeks of age, *n*=3 per group) were anaesthetized as described above. An incision was made in the dorsal skin of the tail, exposing the discs between the tail tendons. An external fixator was attached using 20 G needles, and five discs (C3/4–7/8) were fixed. Using a microscope, the AF of three discs was then cut in a box shape using a 20 G needle and removed while being careful to avoid NP prolapse. A solution of liquefied collagen gel containing C3H10T1/2-V or C3H10T1/2-VM cells (4 × 10^7^ cells per ml) was placed in the cavity and the skin was sutured. After 8 weeks, the mice were killed by cervical dislocation and the discs were excised and processed for histochemistry, IHC and TEM.

### Modified tail-looping model

We modified the tail-looping model that Sakai *et al.* created^38^. C3H/HeSlc mice (male, 10 weeks of age, *n*=5 per group) were anaesthetized and mouse models for transplantation of C3H10T1/2-V or C3H10T1/2-VM cells into the OAF were created as described above. After 8 weeks, these mice were re-anaesthetized, external fixators were removed and the tails were looped at a fixed position between vertebrae 2 and 9 using a 0.88 mm stainless steel wire. The fixed distance of vertebrae 2 and 9 was unified to 5 mm. The distal tail end was excised. As a control group, the same treatment was applied to C3H/HeSlc mice (male, 18 weeks of age, *n*=5) with the AF intact. After 4 weeks, the mice were killed by cervical dislocation and the discs were excised and processed for histochemistry. C5/6 was analysed. To evaluate statistical differences in this study, a minimum size ofive 5 mice per group is needed. Mice were allocated to each group randomly.

### Histochemistry and IHC

For histochemistry, mouse spines were obtained after killing by cervical dislocation (E14.5, 18.5, P1 and 4 weeks of age: C57BL/6N; *n*=3, 10 weeks, 6 months, 12 months and 21 months of age: three groups of littermates, wild type; *n*=4–5, *Mkx*^−/−^; *n*=5, 18 weeks of age: C3H/HeSlc, *n*=3 per group). The samples were fixed in 4% paraformaldehyde (PFA) in PBS for 2 days and decalcified with 10% EDTA/PBS (pH=7.1–7.2) for 14 days. The samples were then processed for paraffin sectioning (10 μm) and stained with Safranin O fast green stain (Wako) or Masson's trichrome stain (Promega).

For IHC, mouse spines were obtained after killing by cervical dislocation (E14.5, E18.5, P1, P4 and10-week-old *Mkx*^+/−^, 18-week-old C3H/HeSlc, 10-week-old C57BL6/N or *Mkx*^−/−^, 21-month-old *Mkx*^+/+^ or *Mkx*^−/−^). The samples were fixed in 4% PFA/PBS for 2–2.5 h and processed for frozen sectioning (5–10 μm). The sections were incubated with 10% EDTA/PBS (pH 7.1–7.2) for 1 h (GFP and Collagen 1), PBS for 10 min (Collagen 14, Bgn and CD24) or 0.1% Tween 20/PBS for 10 min (KRT18). The samples were blocked in Blocking One solution (Nacalai Tesque) for 30–60 min, and then incubated with an anti-GFP antibody (1:500, MBL), anti-Collagen 1 antibody (1:500, ab34710, Abcam), anti-Collagen 14 antibody (1:100, LS-C119470, LSBio), anti-Bgn antibody (1:100, LS-C341858, LSBio), anti-CD24 antibody (1:100, 553262, Bioscience) and anti-KRT18 antibody (1:250, ab668, Abcam) overnight at 4 °C. The sections were then incubated with an Alexa Fluor 488 donkey anti-rabbit antibody (1:500, Life Technologies), an Alexa Fluor 594 donkey anti-rabbit antibody (1:500; Life Technologies), an Alexa Fluor 594 donkey anti-mouse antibody (1:500; Life Technologies) or Hoechst 33342 (1:2,000; Life Technologies) for 1 h. The sections for Collagen 14, Bgn, CD24 and KRT18 were mounted with VECTASHIELD Antifade Mounting Medium with 4,6-diamidino-2-phenylindole (H-1200, Vector Laboratories) without Hoechst 33342 incubation.

Human spines were obtained (nine discs from five humans; Supplementary Fig. 1a) and processed for paraffin sectioning (2 μm). All discs were used for IHC analyses. The sections were first activated with 3% H_2_O_2_ for 10 min and with 1% citric acid buffer at 98–100 °C for 10 min in a microwave oven. The sections were then blocked with Blocking One solution (Nacalai Tesque) for 60 min and incubated with a rabbit anti-human MKX antibody (1:500, LS-C30267; LifeSpan Biosciences) overnight at 4 °C. The sections were then incubated with a rabbit Alexa Fluor 488 donkey anti-rabbit antibody (1:500; Life Technologies) and Hoechst 33342 (1:2000; Life Technologies) for 1 h. Confirmable signals in 10 × views were recorded in three fields per view, and the sum and percentage of Hoechst-positive MKX-positive cells were calculated.

### *In situ* hybridization

Mouse embryos were obtained after euthanasia by cervical dislocation (E14.5 of *Mkx*^*+/−*^ mice). The samples were fixed overnight in 4% PFA/PBS and processed for frozen sectioning (16 μm). Sections were treated with 10 μg ml^−1^ proteinase K (Roche) for 10 min at room temperature (RT) and then acetylated with acetylation solution for 20 min at RT. Pre-hybridization (in × 5 SSC (pH 4.5), 50% formamide, 1% SDS, 50 μg ml^−1^ yeast tRNA (Roche) and 50 μg ml^−1^ heparin (Nacalai Tesque)) was performed at 68 °C for 2 h; then, a DIG–RNA probe (500 ng ml^−1^) was added and hybridized for 14 h at 68 °C. Subsequently, sections were subjected to a series of post-hybridization washes in wash buffers containing formamide, SSC, SDS and 0.05% CHAPS. After blocking with 2% BM Blocking reagent (Roche) containing 0.1% Tween 20 (TBST) for 30 min at RT, embryos were incubated with anti-DIG-AP Fab antibody fragments (Roche) and 1% sheep serum in TBST for 3 h at RT. After a series of washes with TBST, embryos were equilibrated with NTMT (5 M NaCl, 1 M Tris–HCl (pH 9.5), 1 M MgCl_2_ and 0.1% Tween 20). Colour development reactions were performed at 4 °C or RT with nitro blue tetrazolium/5-bromo-4-chloro-3-indolyl phosphate (Roche).

### Electron microscopy analysis

Lumbar discs (L4/5 level) from Mkx^−/−^ or wild-type mice (10 weeks of age, male, one per group of littermates) and tail discs from 8-week postoperative C3H/HeSlc mice (18 weeks of age, *n*=3 per group) were dissected and fixed in 2.5% glutaraldehyde solution for 2 h and 1% osmium solution (Sigma-Aldrich) for 1 h. After fixation, tissues were processed for sectioning (1 μm).

For scanning electron microscopy, the specimens were dried in a critical point drying apparatus (HCP-2; Hitachi, Tokyo, Japan) with liquid CO_2_ and were spatter-coated with platinum, and then examined by scanning electron microscopy (S-4500; Hitachi, Tokyo, Japan, voltage: 15 kV, current: 10 μA and resolutions:1.5 nm).

For TEM, the specimens were embedded in Epon 812. Sliced at ultrathin 90 nm, it were collected on copper grids and were double-stained with uranyl acetate and lead citrate, and then observed using TEM (H-7100, Hitachi, Tokyo, Japan, voltage: 75 kV, current: 15 μA and resolutions: 0.38 nm).

The Image J software was used for the measurement of collagen fibril diameters (100 collagen fibrils were measured three times using different views. Two blinded investigators measured the results).

### Microcomputerized tomography

To evaluate intact spines, microcomputerized tomography (Aloka Latheta LCT 200; Hitachi, Japan, voltage, 80 kV; current, 0.5 mA; and resolution, 60 μm) was used after the mice were killed by cervical dislocation (10 weeks, and 6, 12 and 21 months of age: three groups of littermates, wild type; *n*=4–5, *Mkx*^−/−^; *n*=4, the same samples that were used for histochemistry). The scan was done in air.

### Chondrogenic and osteogenic differentiation

C3H10T1/2, C3H10T1/2-V and C3H10T1/2-VM cells were stimulated for chondrogenic and osteogenic differentiation. Chondrogenic differentiation was performed as follows. Trypsinized cells were resuspended in α-MEM (Sigma-Aldrich) at a concentration of 2 × 10^7^ cells per ml^−1^, and a 20 μl drop of this cell suspension was placed in the centre of a well in a 12-well plate (Cellstar; Greiner Bio-One). The cells were allowed to adhere for 2 h at 37 °C and 5% CO_2_, and 1 ml of α-MEM (Sigma-Aldrich) containing BMP2 (100 ng ml^−1^) was added to the culture. Chondrogenic differentiation was analysed by alcian blue staining, acidic mucopolysaccaride quantification and qRT–PCR after 9 days of culture. Acidic mucopolysaccaride quantification was performed using the Acidic Mucopolysaccaride Assay kit (AK03, Cosmo Bio). Osteogenic differentiation was performed as follows. Trypsinized cells were resuspended in α-MEM (Sigma-Aldrich) at 8 × 10^4^ cells per well in a 12-well plate (Cellstar; Greiner Bio-One). The cells were incubated for 24 h, after which the medium was replaced with the induction medium (α-MEM, 10% FBS, 10 nM dexamethasone, 50 μM ascorbic acid, 10 mM β-glycerophosphate and 100 ng ml^−1^ of BMP2). Osteogenic differentiation was analysed by alizarin red staining, calcification evaluation and qRT–PCR after 14 days of culture. Calcification evaluation was performed using the Calcification Evaluation Set (CSR-ARD-SET; Cosmo Bio). PBS-washed cells were fixed with 4% PFA/PBS and then stained with Alizarin Red Solution (CSR-ARD-A1; Cosmo bio). After washing the cells, the alizarin red dye was extracted with Calcified Nodule Extraction Solution (CSR-ARD-E1; Cosmo Bio), and the absorbance at 450 nm was determined using a microplate reader.

### Adipogenic differentiation

Trypsinized cells were resuspended in medium and added at 1.2 × 10^4^ per well in a 12-well plate. They were cultured until they reached 100% confluency, and then placed in induction medium (α-MEM, 10% FBS, 1 μM dexamethasone, 0.5 mM IBMX and 10 μg ml^−1^ insulin). After 3 days, the medium was changed to maintenance medium (α-MEM, 10% FBS and 10 μg ml^−1^ insulin). Two mediums were then changed alternately every 3 days. Adipogenic differentiation was analysed by microscopy and qRT–PCR after 14 days of culture.

### Alcian blue staining

The cells were first fixed in 4% PFA/PBS for 10 min, then incubated in 0.1 N HCl for 5 min and exposed to 0.5% Alcian blue 8GX/0.1 N HCl (pH 1.0) for 3 h. After destaining with 0.1 N HCl, the cells were evaluated for Alcian blue staining using a stereomicroscope. The samples were prepared from three independent clones from each condition.

### Alizarin red staining

The cells were first fixed in 4% PFA/PBS for 30 min, then exposed to 2% Alizarin red S (Sigma-Aldrich) for 5 min. After washing with diluted water, the cells were evaluated for Alizarin red staining. The samples were prepared from three independent clones from each condition.

### Quantitative real-time PCR

Total RNA was extracted from cell cultures (OAF cells of C57/BL6N, OAF cells of *Mkx*^−/−^, HAF, C3H10T1/2, C3H10T1/2-V, C3H10T1/2-VM and cells after two mesodermal differentiations). qRT–PCR was performed using SYBR Green PCR Master Mix (Applied Biosystems). The primer sequences used for qRT–PCR are listed in the Supplementary Table 2 (mouse) and Supplementary Table 3 (human). The results are expressed as messenger RNA (mRNA) levels corrected to GAPDH levels in each sample. Total RNAs were prepared at least three independent cell samples from each experiment.

### Microarray analysis

DNA microarray analysis was performed using Affymetrix mouse genome 430 2.0 array. RNA samples were obtained from C3H10T1/2, C3H10T1/2 overexpressing Venus and C3H10T1/2 overexpressing Venus-Mohawk. Total RNA (200 ng) was reverse-transcribed and biotinylated using the GeneChip 3′ IVT Express kit (Affymetrix). The microarray data were summarized using the MAS 5.0 method. Microarray data that support the findings of this study have been deposited in GEO with the primary accession code GSE81276.

### Western blotting

Whole-cell proteins were extracted from C3H10T1/2-V and C3H10T1/2-VM cells, and HAF cells. Cellular proteins collected in SDS–PAGE sample buffer were electrophoresed on 4–12% SDS-bis-ris gels and transferred to polyvinylidene difluoride membranes. Membranes were blocked in Blocking One solution (Nacalai Tesque) for 30–60 min. After blocking, the membranes were incubated overnight at 4 °C with dilutions of anti-Smad1 (1:500, #9743; Cell Signaling Technology), anti-phospho-Smad1/5/8 (1:500, #9511; Cell Signaling Technology), anti-Smad2/3 (1:500 BD #610843), anti-phospho-Smad3 (1:1,000 Abcam Ab52903), anti-Sox9 (1:1,000 Millipore AB5535), anti-Bgn (1:1,000 LSBio LS-C341858), anti-Col1 (1:250 Abcam ab21286), anti-Dcn (1:250 Abcam ab137505), anti-β-actin (1:1,000 SIGMA A5316) and anti-GAPDH (1:2,000, MAB374; Millipore) antibodies. Rinsed membranes were incubated for 1 h with ECL mouse or rabbit IgG, horseradish peroxidase-Linked Whole antibody (GE Healthcare). The enhanced chemiluminescence immunoblotting detection system (Thermo Scientific) was used to detect the antigen–antibody complexes. Extracts were prepared from three independent samples. Full scans of the key immunoblots are indicated to Supplementary Fig. 12.

### Luciferase assay

C3H10T1/2-V or C3H10T1/2-VM cells were seeded at 1 × 10^4^ cells per well in 96-well plates (Cellstar; Greiner Bio-One). On the following day, the ID1-Bre luciferase construct (Addgene)^35^ was transfected using FugeneHD (Promega). Twenty-four hours later, the medium was changed to α-MEM plus 0.5% FBS and 1% PS. Twelve hours later, BMP2 at 100 ng ml^−1^ was added to the medium. Ten hours later, luciferase activity was quantified using the luciferase assay (Promega) on a luminometer (ARVO × 3; PerkinElmer). The assay was performed three times.

### Scoring system of IVD degeneration

We used the IVD degenerative histological score^30^ to evaluate the whole IVD of ageing wild-type and *Mkx*^−/−^ mice. Two blinded orthopedic surgeons performed each image evaluation and each scoring.

To evaluate the AF of modified tail-looping model mice, we used the Nishimura system^39^, a histological grading system of degenerative changes in the AF. This system is classified into six grades (grade 0: normal structure; grade 1: mildly serpentine with rupture; grade 2: moderately serpentine with rupture; grade 3: severely serpentine with mildly reversed contour; grade 4: severely reversed contour; grade 5: indistinct).

### Statistical analysis

Data are presented as the mean±s.e.m. The Student's *t*-test was used to evaluate differences between groups. A one-tailed *t*-test was performed for Figs 6e and 8l,o, while a two-tailed *t*-test was performed for the other figures. *P* values<0.05 were considered significant. *P<0.05. **P<0.01. ***P<0.001.

### Data availability

Microarray data that support the findings of this study have been deposited in GEO with the primary accession code GSE81276. The authors declare that all other data supporting the findings of this study are available within the article and its files.

## Additional information

**How to cite this article:** Nakamichi, R. *et al.* Mohawk promotes the maintenance and regeneration of the outer annulus fibrosus of intervertebral discs. *Nat. Commun.* 7:12503 doi: 10.1038/ncomms12503 (2016).

## Supplementary Material

Supplementary Information Supplementary Figures 1-12 and Supplementary Tables 1-3.
