# Supplementary material for: Mohawk promotes the maintenance and regeneration of the outer annulus fibrosus of intervertebral discs
Source: Nat Commun. 2016 Aug 16;7:12503. doi: 10.1038/ncomms12503 (PMC4990710; doi:10.1038/ncomms12503)
Supplement: Supplementary Information — Supplementary Figures 1-12 and Supplementary Tables 1-3. [file ncomms12503-s1.pdf]

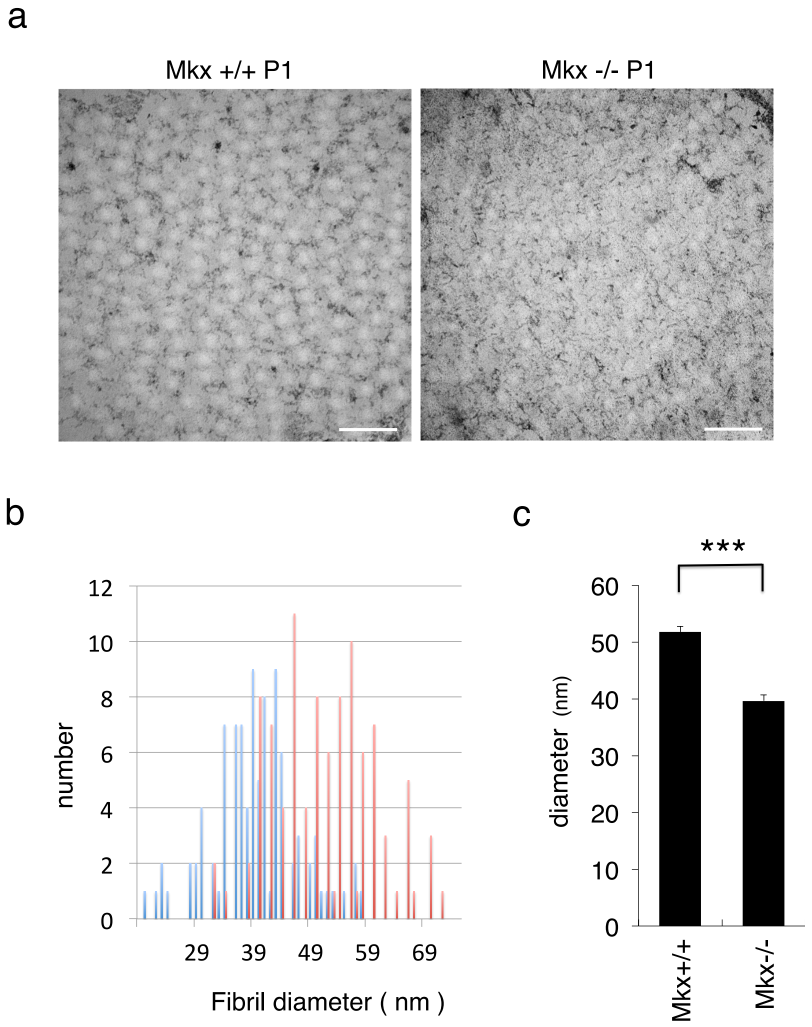

Supplementary Figure 1 | Scale analyses of the annulus fibrosus (AF) from *Mkx*$^{-/-}$ mice at postnatal day 1 (P1). (**a**) Images of transmission electron microscopy of the outer annulus fibrosus (OAF) of *Mkx*$^{+/+}$ and *Mkx*$^{-/-}$ mice at P1. Scale bar, 200 nm. (**b**) Histogram of the diameter of collagen fibrils from *Mkx*$^{+/+}$ and *Mkx*$^{-/-}$ mice at P1. Blue bar, *Mkx*$^{-/-}$; red bar, *Mkx*$^{+/+}$. (**c**) The calculated mean of the diameter of 100 collagen fibrils from *Mkx*$^{+/+}$ and *Mkx*$^{-/-}$ mice at P1: for *Mkx*$^{+/+}$, mean diameter = 51.73 nm (SD = 7.57 nm); for *Mkx*$^{-/-}$, mean diameter = 39.63 nm (SD = 8.99 nm). Blue bar, *Mkx*$^{-/-}$; red bar, *Mkx*$^{+/+}$. Error bars represent the SD. *** $P < 0.001$. SD, standard deviation. Statistical differences were assessed with Student's *t*-test.

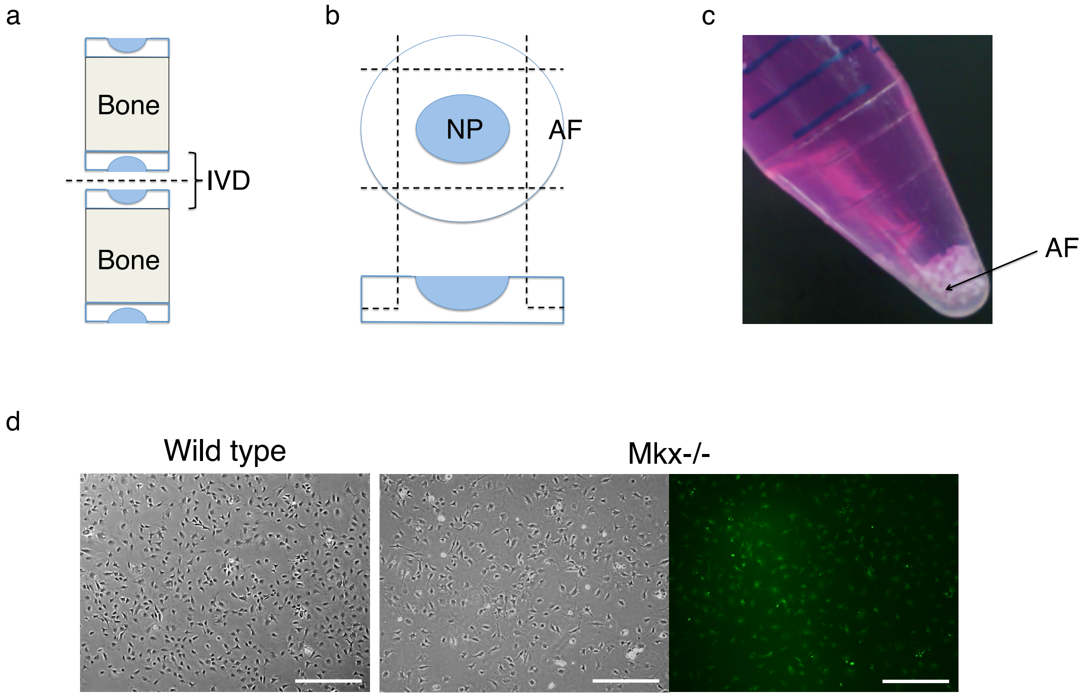

Supplementary Figure 2 | Detailed method for isolation of annulus fibrosus (AF) cells. (**a**) Schema for cutting off the mouse tail. (**b**) Schema for dissecting pieces of the AF. (**c**) AF pieces in medium. (**d**) Macroscopic view of the cultured AF cells of *Mkx*$^{+/+}$ and *Mkx*$^{-/-}$ mice. The right view is a fluoroscopic view of AF cells from a *Mkx*$^{-/-}$ mouse. Scale bar, 500 μm.

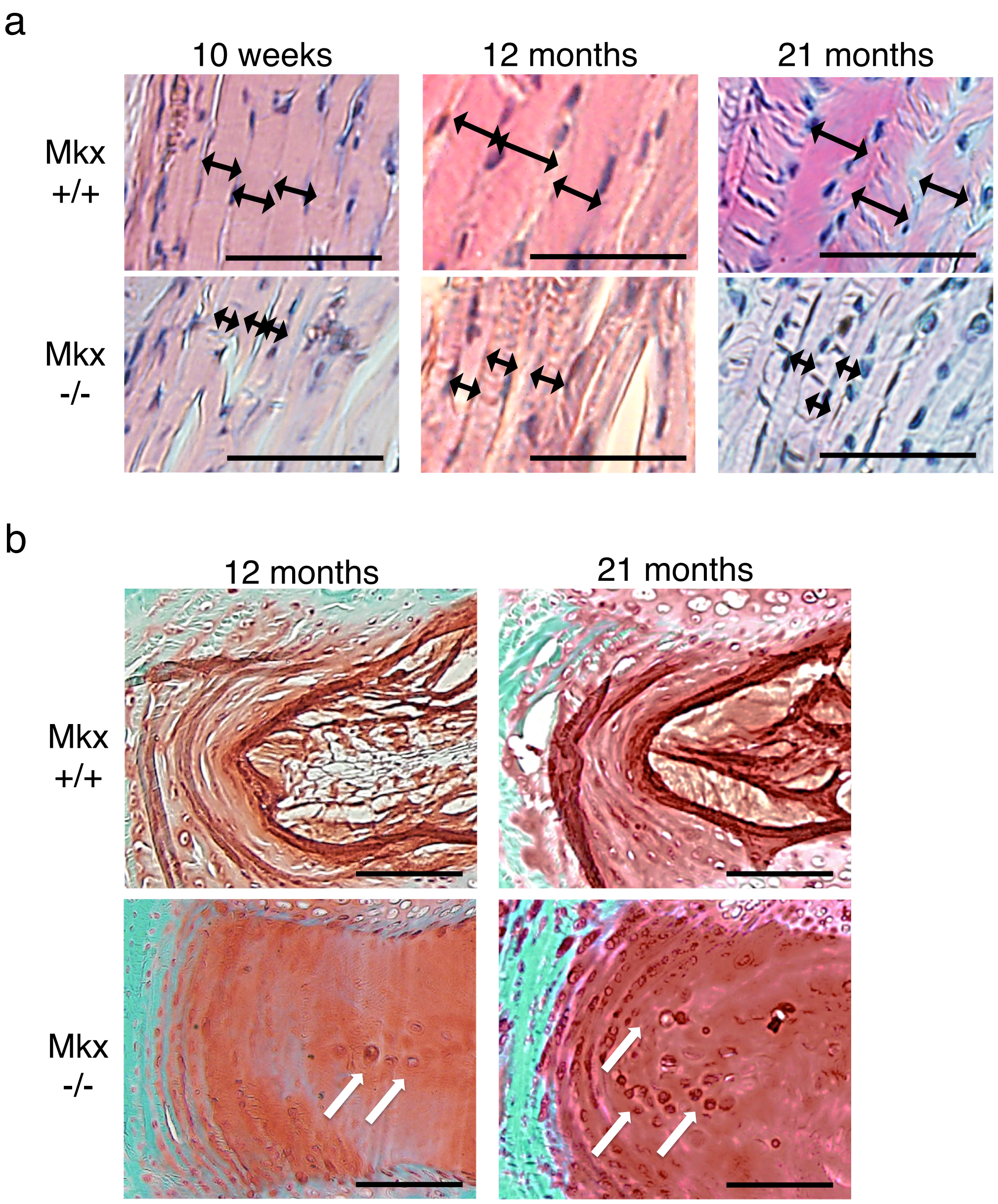

Supplementary Figure 3 | The intervertebral discs in *Mkx*$^{-/-}$ mice at various stages. (**a**) HE staining of outer annulus fibrosus (OAF) of L3/4 at 10, 12, and 21 months. Yellow allow indicates the width of the OAF collagen fiber. Scale bars, 100 μm. (**b**) Safranin-O-fast green staining of sagittal sections of L3/4 at 12 and 21 months. White arrow indicates small round cells resembling chondrocytes. Scale bars, 300 μm.

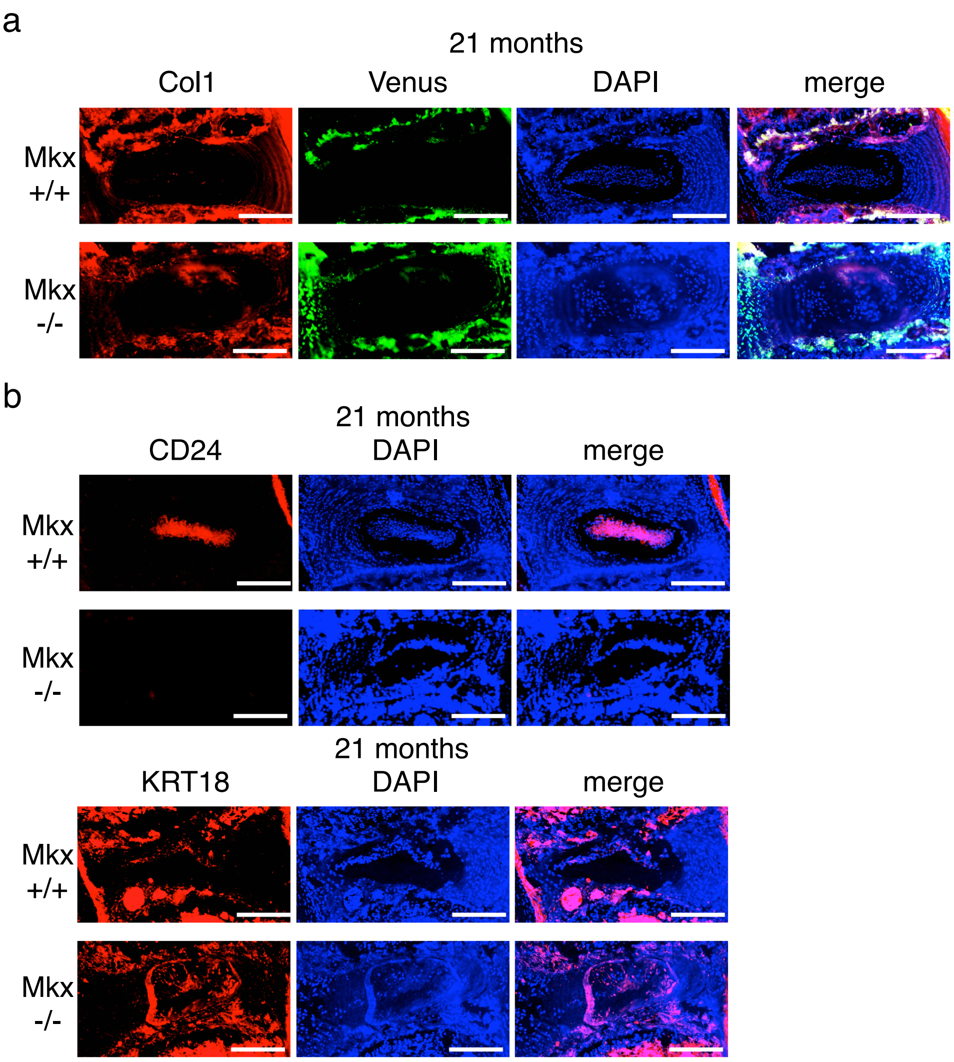

Supplementary Figure 4 | The expression of gene markers of aged *Mkx*$^{+/+}$ and *Mkx*$^{-/-}$ mice. (**a, b**) Immunohistochemistry shows that Col1, Venus and CD24 were negative and KRT18 was positive in mutant nucleus pulposus of 21-month-old *Mkx*$^{-/-}$ mice. Blue: DAPI, Green: Venus, Red: Col1, CD24 and KRT18. Scale Bars, 500 µm.

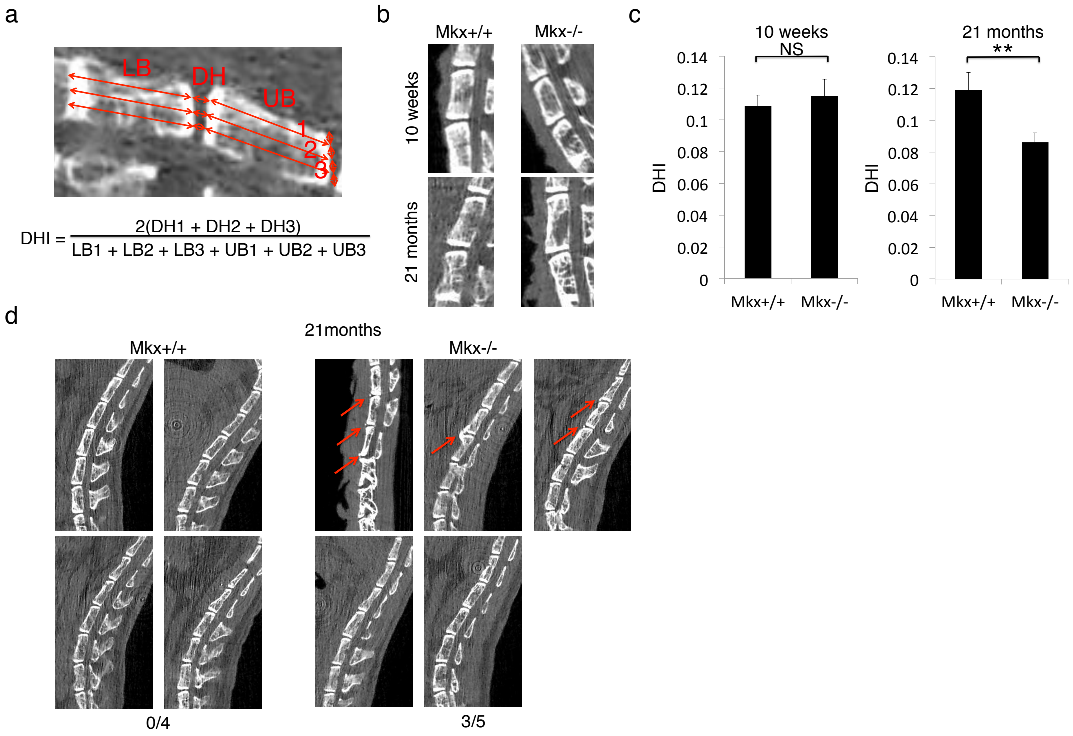

Supplementary Figure 5 | Computerized tomography (CT) analyses of *Mkx*$^{+/+}$ and *Mkx*$^{-/-}$ mice. (**a**) Methods for measurement of the lengths between L6 and S1 (L6/S1) and for calculation of the disc height index (DHI). (**b**) Sagittal CT images of the lumbar spine at 10 weeks and 21 months. (**c**) DHI scores of L6/S1 in *Mkx*$^{+/+}$ and *Mkx*$^{-/-}$ mice. (**d**) Sagittal CT images of the lumbar spine at 21 months of *Mkx*$^{+/+}$ and *Mkx*$^{-/-}$ mice. Red arrows indicate bone spur. DH: disc height. UB: upper body. LB: lower body. DHI: disc height index. ** $P < 0.01$. NS: not significant. Statistical differences were assessed with Student's *t*-test.

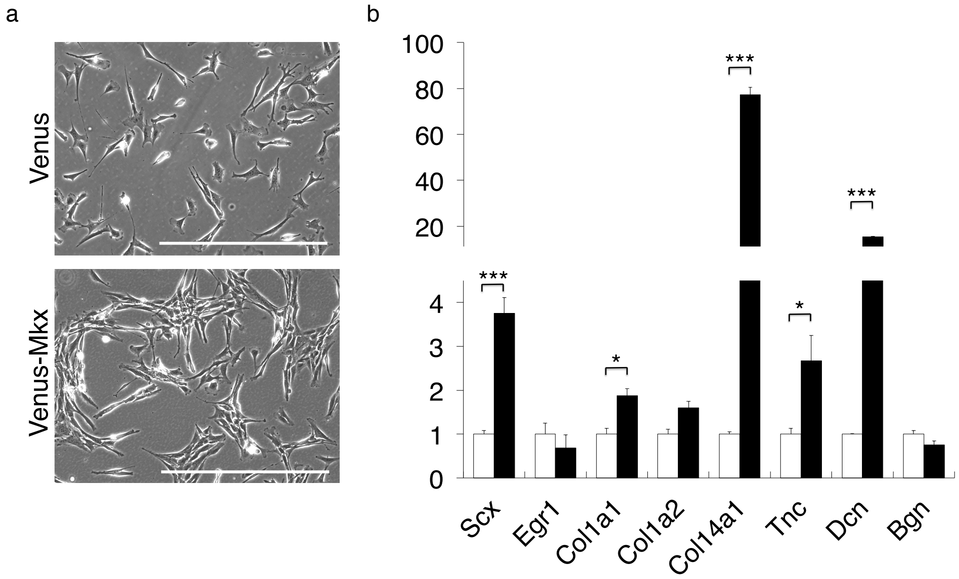

Supplementary Figure 6 | *Mkx* promotes the differentiation of mouse bone marrow derived mesenchymal stem cells (MBMMSCs) to ligament cells. (**a**) Images of BMMSCs induced by Venus (BMMSCs-V) and Venus-Mkx (BMMSCs-VM) in plate culture. Scale bars, 500 μm. (**b**) Quantitative real-time polymerase chain reaction analyses of the expression of ligament-related genes in BMMSCs-V and BMMSCs-VM. mRNA levels in BMMSCs-V were normalized to 1. Error bars represent Error bars represent s.e.m. * $P < 0.05$, *** $P < 0.001$. Statistical differences were assessed with Student's *t*-test.

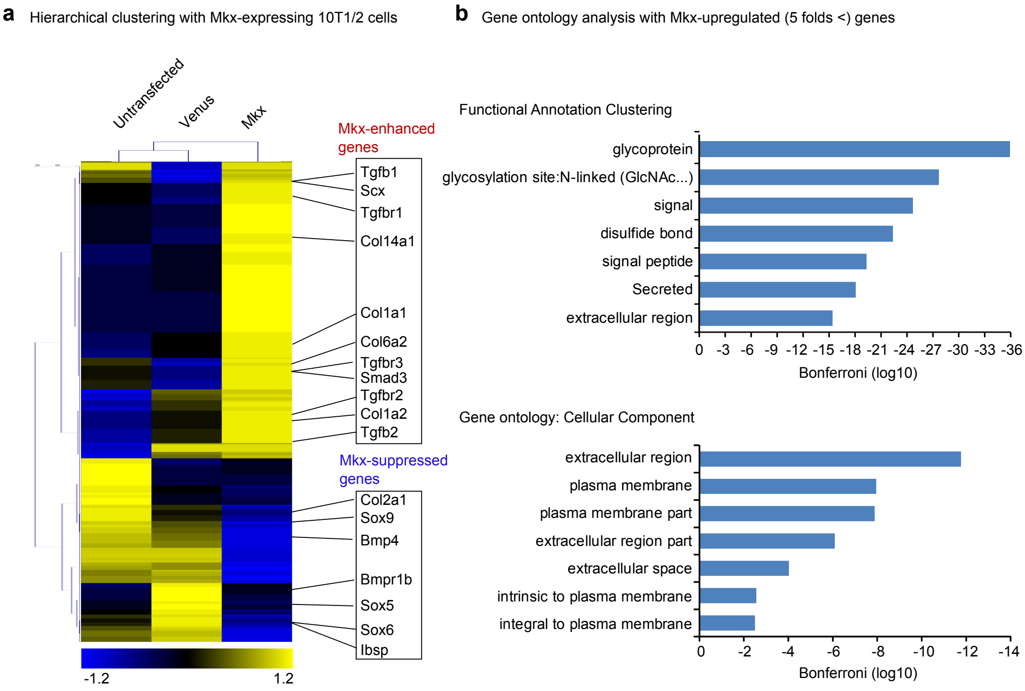

Supplementary Figure 7 | Microarray analyses of three C3H10T1/2 cells. (**a**, **b**) Microarray analyses of C3H10T1/2 and C3H10T1/2 cells induced by Venus (C3H10T1/2-V) or Venus-Mkx (C3H10T1/2-VM). Microarray analyses revealed that C3H10T1/2-VM cells acquired beneficial changes in the differentiation of outer annulus fibrosus cells.

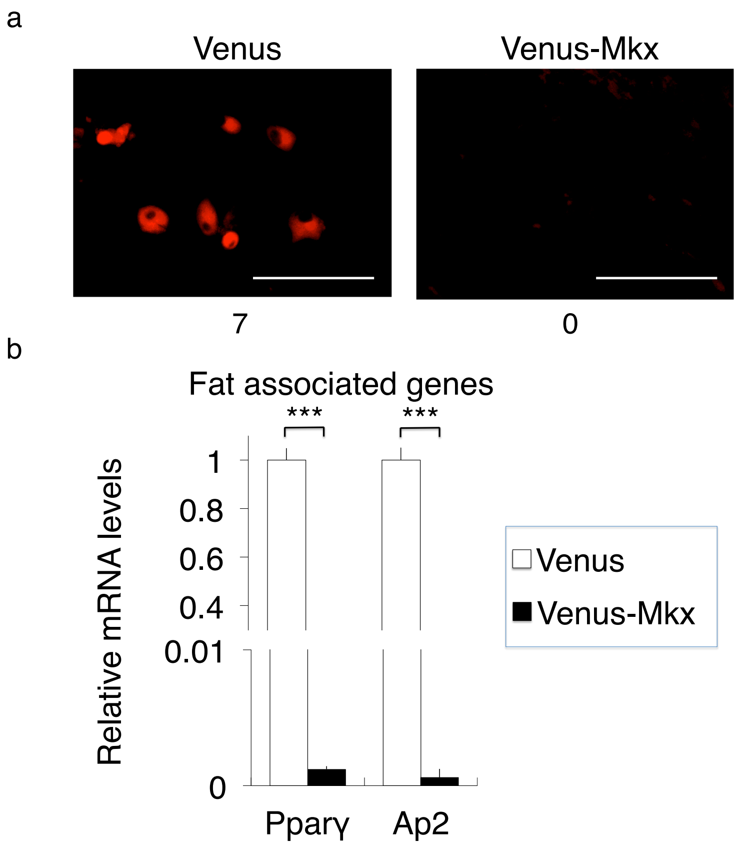

Supplementary Figure 8 | Adipogenic differentiation was severely disrupted in C3H10T1/2-VM cells. (**a**) AdipoRed staining of C3H10T1/2-V and C3H10T1/2-VM cells after induction of adipocyte differentiation. Scale Bar, 500 nm. (**b**) qRT–PCR analyses of adipogenic gene expression in C3H10T1/2-V and C3H10T1/2-VM cells. mRNA levels in C3H10T1/2-V cells were normalized to 1. Error bars represent Error bars represent s.e.m. *** $P < 0.001$. Statistical differences were assessed with Student's *t*-test.

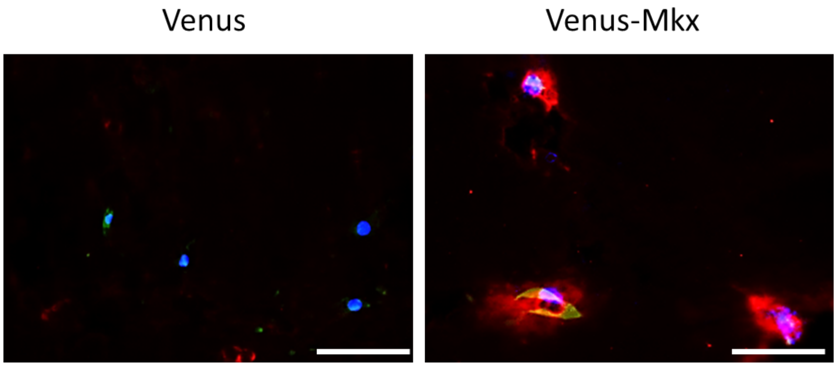

Supplementary Figure 9 | Immunohistochemistry (IHC) of three-dimensional cultured cells. IHC showed high Col1a1 protein expression in C3H10T1/2-VM cells compared with C3H10T1/2-V cells cultured at 8 weeks. Red: Col1a1. Blue: Hoechst. Green: Venus. Scale Bar, 100 nm.

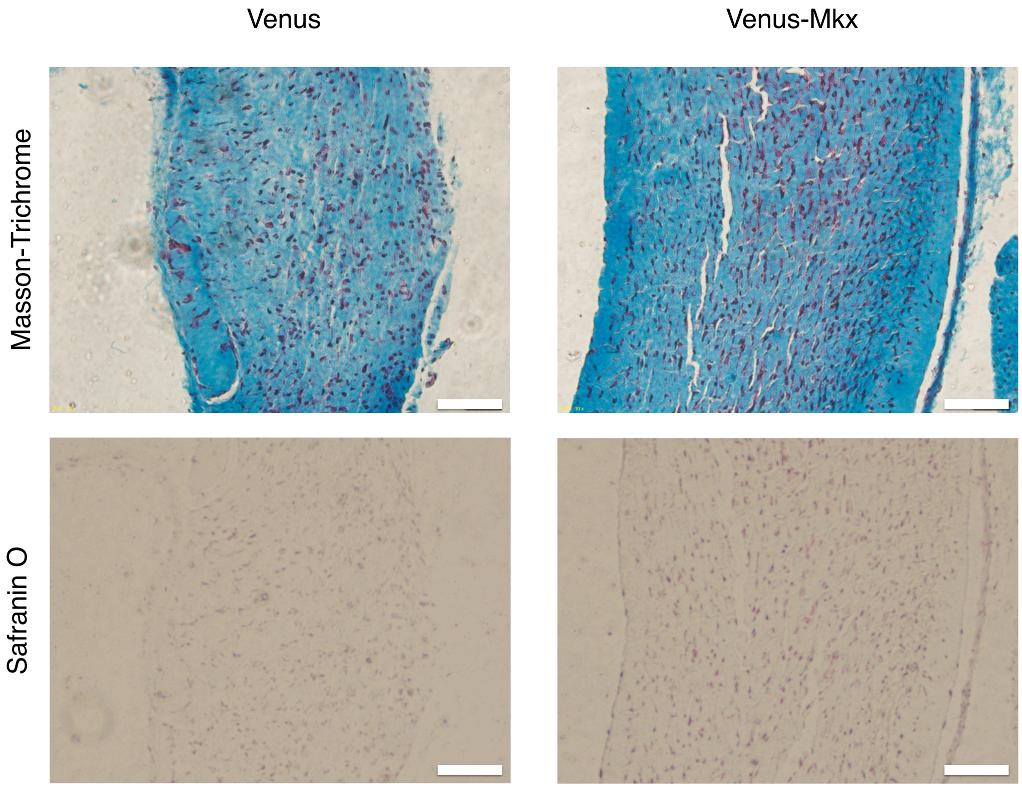

Supplementary Figure 10 | Histological analyses of transplanted gels.
Masson-trichrome and Safranin O staining of type I collagen gels after subcutaneous transplantation in dorsal skin pockets of mice. Scale Bars, 100 µm.

a b

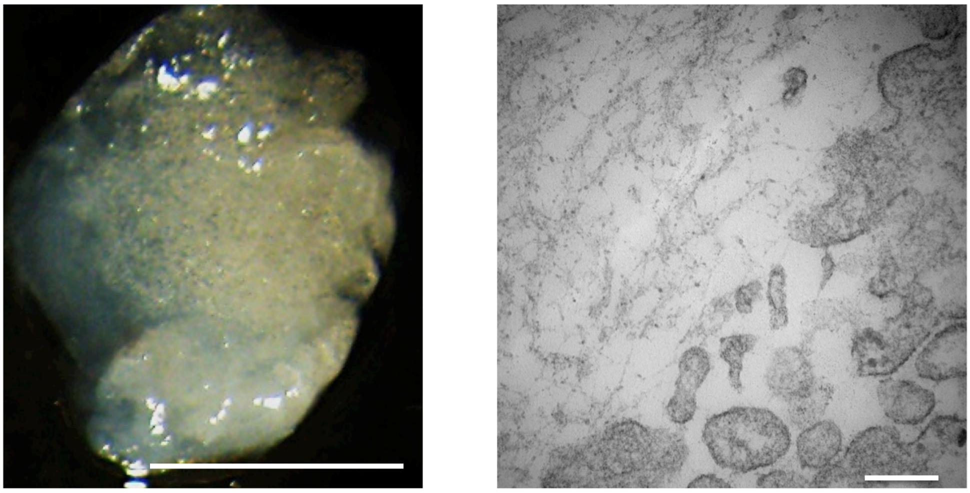

Supplementary Figure 11 | Views that only collagen gel was transplanted. (**a**) Gross appearance of products 8 weeks after subcutaneous transplantation of only collagen gels. Scale bar, 1 mm. (**b**) Images from transmission electron microscopy of the control group with only gel transplanted subcutaneously. Collagen fibrils are difficult to confirm. Scale Bar, 200 nm.

Figure 3d

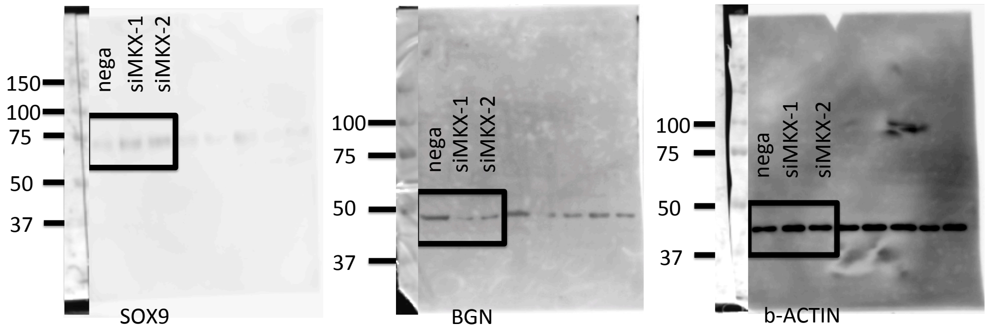

Figure 5f

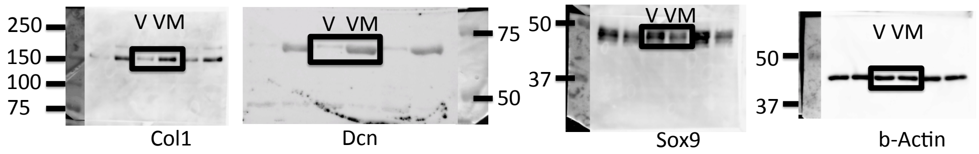

Figure 6b

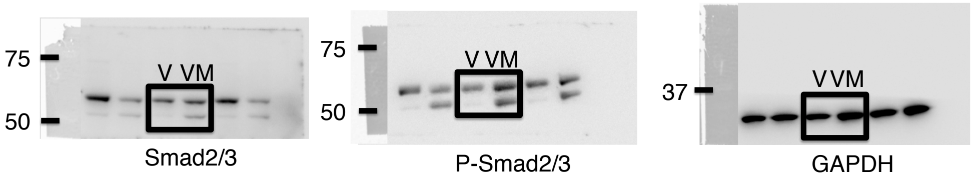

Figure 6d

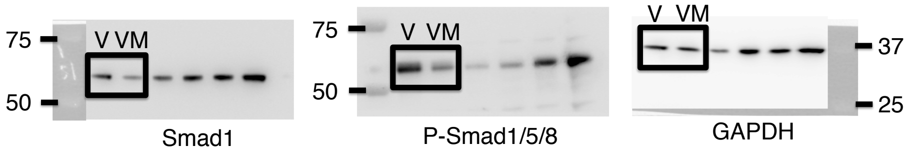

Supplementary Figure 12 | Full scans of the key immunoblots. Boxes indicates the part used in the Figures and numbers indicate the Molecular Weight (KDa).

Supplementary Table 1 | Details of human samples. Lumbar discs were obtained postmortem from five subjects (nine discs).

#	Age	Sex	Level	Thompson grade
1	20	M	L1/2	1.5
	20	M	L2/3	2
	20	M	L4/5	2.5
2	43	F	L3/4	3
	43	F	L4/5	3
3	55	F	L2/3	3
	55	F	L3/4	3
4	52	M	L4/5	4
5	64	M	L4/5	4

Supplementary Table 2 | Primers used for quantitative real-time polymerase chain reaction. Primers were designed from the *mus musculus* genome. Primers reported in the past were adopted partially[50].

gene	Forward primers	Reverse primers
Gapdh	5'-TTGTGGAAGGGCTCATGACC-3'	5'-TCTTCTGGGTGGCAGTGATG-3'
Mkx	5'-GATGGCGACTCCTGCTCTGA-3'	5'-CGGTCTGCCGCCAGCTTTTA-3'
Scx	5'-CCTTCTGCCTCAGCAACCAG-3'	5'-GGTCCAAAGTGGGGCTCTCCGTGACT-3'
Egr1	5'-CAGCGCCTTCAATCCTCAAG-3'	5'-GCGATGTCAGAAAAGGACTCTGT-3'
Tnmd	5'-AACACTTCTGGCCCGAGGTAT-3'	5'-AAGTGTGCTCCATGTCATAGGTTTT-3'
Col1a1	5'-GAGCGGAGAGTACTGGATCG-3'	5'-GCTTCTTTTCCTTGGGGTTC-3'
Col1a2	5'-CCAGCGAAGAACTCATACAGC-3'	5'-GGACACCCCTTCTACGTTGT-3'
Col2a1	5'-TTCCACTTCAGCTATGGCGA-3'	5'-GACGTTAGCGGTGTTGGGAG-3'
Col3a1	5'-CTAAAATTCTGCCACCCCGAA-3'	5'-AGGATCAACCCAGTATTCTCCACTC-3'
Col5a1	5'-CCTGGCATCAACTTGTCCGATGG-3'	5'-GTGGTCACTGCGGCTGAGGAACTTC-3'
Col12a1	5'-CCGTGTTGTGTATCGCCCT-3'	5'-CACCTTAGCAACCATCTGCCTC-3'
Col14a1	5'-GAGCAGAGACCACATTGGCC-3'	5'-CGTACAGCTCGAGGTCGGAA-3'
Tnc	5'-AACCATCAATGCGGCCAC-3'	5'-TGTCGTCCAGAAAAACGTCAGA-3'
Dcn	5'-CTATGTGCCCCTACCGATGC-3'	5'-CAGAACACTGCACCACTCGAAG-3'
Bgn	5'-GGGCGTAGAGGTGCTGGAG-3'	5'-TTTCTGAGCTTCGCAAGGATG-3'
Tnxb	5'-AGTCCAACAGGAAGTATAAGATGAACCTC-3'	5'-TGGTCTACAACATCTGGTGGGG-3'
Fn1	5'-CACGTACCTCTTCAAAGTCTTTGC-3'	5'-GGATTGCTTTCCCTGCCCT-3'
Fbn1	5'-GGACACGATGCGCTGAAAGG-3'	5'-CAGGAATGCCGGCAAATGGG-3'
Eln	5'-CAAGTCGGAGCTGGCATCGG-3'	5'-GTGGGAACTCCAGGGAGCAC-3'
Sox5	5'-GAAGGAGCGGCAGCTCATGG-3'	5'-GTTGCCTCGCGATCTGCTCC-3'
Sox6	5'-GACCTTGCTCGCCAACAGCA-3'	5'-CAAGGTCCGCTGGTCATGTG-3'
Sox9	5'-GTGAAGAACGGACAAGCGGA-3'	5'-CTGAGATTGCCCAGAGTGC-3'
Acan	5'-CAGCAGCACCATCACAGAGT-3'	5'-TTTCTGCTGTCTGGGTCTCC-3'
Runx2	5'-GGTCCCCGGGAACCAA-3'	5'-GGCGATCAGAGAACAAACTAGGTTT-3'
Osx	5'-CCAGCCTCTGGCTATGCAAA-3'	5'-AGGAAATGAGTGAGGGAAGGGT-3'
ALP	5'-CATGACATCCCAGAAAGAC-3'	5'-GTTGTGAGCGTAATCTACC-3'
Pparγ	5'-TCGCTGATGCACTGCCTATG-3'	5'-GAGAGGTCCACAGAGCTGATT-3'
Smad1	5'-GGTCTGCATCAACCCCTACC-3'	5'-GTGCTGAGGATTGTACTCGCTG-3'
Smad5	5'-CCACTATAAGAGAGTGGAGAGTCC-3'	5'-CAGGTTCCTGAACTGAACCAGAAG-3'
Smad8	5'-CTGCATCAACCCATACCATTACCG-3'	5'-CTTCGGAACTTGGCCAGGAG-3'
Smad2	5'-CACCCACTCCATTCCAGAAAACAC-3'	5'-CTTCACTGATATATCCAGGTGGTGG-3'
Smad3	5'-CGTATGAGCTTCGTCAAAGGCTG-3'	5'-CTTGTCAAGCCACTGCAAGGG-3'
Bmpr1a	5'-GCTGTTCGGAGAAATTGGAACTACAG-3'	5'-CCTTACAGAACAAAATCACTTGGCAATGAC-3'
Bmpr2	5'-GGTCTGTGGGAGAAATCAAAAGGG-3'	5'-CTCTTCATAGTGGCACTCTTGGG-3'
Tgfbr1	5'-CCCGGGGGCGAAGGCATTAC-3'	5'-GCTGCCAGCTCCACAGGACC-3′
Tgfbr2	5'-CGTCCCGCTGCAATGC-3′	5'-CGCACCTTGGAACCAAATG-3′

Supplementary Tables 3 | Primers used for quantitative real-time polymerase chain reaction. All primers were designed from the human genome.

gene	Forward primers	Reverse primers
GAPDH	5'-ATGCCTCCTGCACCACCAAC-3'	5'-GCCATCCACAGTCTTCTGGG-3'
MKX	5'-TCAAGGACAACCTCGGCCTG-3'	5'-ACGGGTTGTCACGGTGCTTG-3'
SCX	5'-AGAACACCCAGCCCAAACAG-3'	5'-GGCCACCTCCTAACTGCGAATC-3'
TNMD	5'-ATGGCAAAGAATCCTCCAGAG-3'	5'-GCTTGCTCCCCCAAAACAGG-3'
COL1A1	5'-TCTGCGACAACGGCAAGGTG-3'	5'-TTTCTTGGTCGGTGGGTGAC-3'
COL1A2	5'-AAGGTCCTCCTGGTCCCAGTG-3'	5'-ATGACGCCAGCTCTGCCATC-3'
COL3A1	5'-CCAGGGAAAGATGGCCCAAG-3'	5'-CAGCTATACCTGGAAGTCCGG-3'
COL5A1	5'-GACTGTGACACCGCAGTACC-3'	5'-GTCTTCGTAGTAGGGGTATTCGTAG-3'
COL12A1	5'-GTGAACCTGTCACTGTTCGGG-3'	5'-CAGGCACAGGTTCCCAAAAGAC-3'
CO14A1	5'-CATCATGCTTCGTGGCTATGGC-3'	5'-GATCTGTCTTGAAGTTGGGTCCC-3'
TNC	5'-AAGCAACCCAGCCAAAGAGACC-3'	5'-CAATAGCTGCCTTGCCATTCCTC-3'
DCN	5'-GGGCTGGCAGAGCATAAGTAC -3'	5'-GGCAGAGCGCACGTAGACAC -3'
BGN	5'-GACAACAACAAGTTGGCCAGGG-3'	5'-GGGACAGAAGTCGTTGACACC-3'
TNXB	5'-CTATGAGGTGACCGTGGTCTC-3'	5'-CCCTCGGTCAAGTTCAGTGC-3'
FN1	5'-CACATTTCCAAGTACATTCTCAGGTGG-3'	5'-GGCTTCAGGCCTTTGATGGTG-3'
FBN1	5'-GCAACAAAGGGTTCCAGCTGG-3'	5'-GTGTACGAACCCTGGTTGTTAATACAC-3'
ELN	5'-CAGTTGTCCCAGGTGCTGGG -3'	5'-CCCAGCTCCAACCCCGTAAG -3'
SOX5	5'-ATGGAAGAGCTCATCAAAAACGAGCC-3'	5'-GTTCCCCGATCCCATTGCAAG-3'
SOX6	5'-ACGGCAGCAAATGGACCTTGC-3'	5'-GGATCTGTTGCTGCAGGAGATTAATTT TG-3'
SOX9	5'-AAGACGCTGGGCAAGCTCTG-3'	5'-GTAATCCGGGTGGTCCTTCTTG-3'
ACAN	5'-CTACGAAGACGGCTTCCACC-3'	5'-CTCATCCTTGTCTCCATAGCAGC-3'
